# Reticulate leaves and stunted roots are independent phenotypes pointing at opposite roles of the phosphoenolpyruvate/phosphate translocator defective in *cue1* in the plastids of both organs

**DOI:** 10.3389/fpls.2014.00126

**Published:** 2014-04-08

**Authors:** Pia Staehr, Tanja Löttgert, Alexander Christmann, Stephan Krueger, Christian Rosar, Jakub Rolčík, Ondřej Novák, Miroslav Strnad, Kirsten Bell, Andreas P. M. Weber, Ulf-Ingo Flügge, Rainer E. Häusler

**Affiliations:** ^1^Department of Botany II, Cologne Biocenter, University of CologneCologne, Germany; ^2^Lophius BiosciencesRegensburg, Germany; ^3^Quintiles GmbHNeu-Isenburg, Germany; ^4^Lehrstuhl für Botanik, Wissenschaftszentrum Weihenstephan, Technische Universität MünchenMunich, Germany; ^5^Institut für Biochemie der Pflanzen, Heinrich-Heine-Universität DüsseldorfDüsseldorf, Germany; ^6^Laboratory of Growth Regulators, Centre of the Region Haná for Biotechnological and Agricultural Research, Institute of Experimental Botany, Palacký UniversityOlumouc, Czech Republic; ^7^Cluster of Excellence on Plant SciencesDüsseldorf, Germany

**Keywords:** secondary metabolism, phosphate translocator, phosphoenolpyruvate, plastids, reticulate mutants

## Abstract

Phosphoenolpyruvate (PEP) serves not only as a high energy carbon compound in glycolysis, but it acts also as precursor for plastidial anabolic sequences like the shikimate pathway, which produces aromatic amino acids (AAA) and subsequently secondary plant products. After conversion to pyruvate, PEP can also enter *de novo* fatty acid biosynthesis, the synthesis of branched-chain amino acids, and the non-mevalonate way of isoprenoid production. As PEP cannot be generated by glycolysis in chloroplasts and a variety of non-green plastids, it has to be imported from the cytosol by a phosphate translocator (PT) specific for PEP (PPT). A loss of function of PPT1 in *Arabidopsis thaliana* results in the c*hlorophyll a/b binding protein underexpressed1* (*cue1*) mutant, which is characterized by reticulate leaves and stunted roots. Here we dissect the shoot- and root phenotypes, and also address the question whether or not long distance signaling by metabolites is involved in the perturbed mesophyll development of *cue1*. Reverse grafting experiments showed that the shoot- and root phenotypes develop independently from each other, ruling out long distance metabolite signaling. The leaf phenotype could be transiently modified even in mature leaves, *e.g*. by an inducible *PPT1*RNAi approach or by feeding AAA, the cytokinin *trans*-zeatin (*t*Z), or the putative signaling molecule dehydrodiconiferyl alcohol glucoside (DCG). Hormones, such as auxins, abscisic acid, gibberellic acid, ethylene, methyl jasmonate, and salicylic acid did not rescue the *cue1* leaf phenotype. The *low cell density1* (*lcd1*) mutant shares the reticulate leaf-, but not the stunted root phenotype with *cue1*. It could neither be rescued by AAA nor by *t*Z. In contrast, *t*Z and AAA further inhibited root growth both in *cue1* and wild-type plants. Based on our results, we propose a model that PPT1 acts as a net importer of PEP into chloroplast, but as an overflow valve and hence exporter in root plastids.

## Introduction

The *Arabidopsis thaliana cue1* mutant has been isolated almost 20 years ago in a screen for mutants defective in light-triggered activation of gene expression during germination (Li et al., 1995). Among the eight so far described *cue* mutants (Lopez-Juez et al., 1998) *cue1* is unique in that the primary defect is not directly involved in light signaling (Vinti et al., 2005), but is caused by a mutation in a phosphate translocator (PT) of the inner envelope membrane of plastids (Streatfield et al., 1999). The phosphoenolpyruvate (PEP)/PT (Fischer et al., 1997) defective in *cue1* belongs, together with the triose phosphate/PT (TPT; Flügge et al., 1989), the glucose 6-P/PT (GPT; Kammerer et al., 1998), and the xylulose 5-P/PT (XPT; Eicks et al., 2002) to the PT gene family (Knappe et al., 2003a). The PPT catalyzes transport of three carbon compounds containing a phosphate group at the C2 position, such as phosphoenolpyruvate (PEP) or 2-phosphoglycerate (2-PGA) (Fischer et al., 1997), whereas, for instance, the TPT transports C3 compounds phosphorylated at the C3 position, such as triose phosphates (TP) or 3-PGA (Fliege et al., 1978). The proposed role of the PPT in C3 plants is the provision of PEP for the shikimate pathway (Fischer et al., 1997), which is entirely localized in the plastid stroma (Herrmann, 1995; Schmid and Amrhein, 1995; Herrmann and Weaver, 1999). As end products the shikimate pathway produces aromatic amino acids (AAA) and a number of downstream products, such as anthocyanidin, flavonoids, the aromatic rings of plastoquinone or tocopherol, and a vast amount of secondary plant products including lignin (Maeda and Dudareva, 2012). Chloroplasts and most other non-green plastids lack the ability to produce PEP by a complete glycolysis (Stitt and Ap Rees, 1979; Journet and Douce, 1985; Prabhakar et al., 2009) and thus rely on the provision of PEP from the cytosol. In most C3 plants, PEP is also imported as precursor of fatty acid biosynthesis (Ohlrogge and Jaworski, 1997; Schwender and Ohlrogge, 2002) and to generate pyruvate for the mevalonate-independent way (2-C-methyl-D-erythritol 4-phosphate [MEP] pathway) of isoprenoid biosynthesis (Lichtenthaler, 1999; Phillips et al., 2003), for example during isoprene emission (e.g., Li and Sharkey, 2013). In contrast, in C4 and Crassulaceen acid metabolism (CAM) plants the PPT acts as net PEP exporter from the chloroplasts. During C4 photosynthesis and CAM, PEP is synthesized from pyruvate in the mesophyll chloroplasts by pyruvate:phosphate dikinase (PPDK) and has to be exported to the cytosol, where PEP carboxylase, the starting point of the C4 cycle or CAM, is localized (e.g., Häusler et al., 2000; Langdale, 2011).

The *cue1* mutant is characterized by reticulate leaves, based on smaller mesophyll cells compared to the bundle sheath cells and mesophyll cells of the wild type (Li et al., 1995). In line with the proposed physiological role of the PPT, the *cue1* mutant has been shown to contain less AAA in leaves and a selective reduction of secondary products (Streatfield et al., 1999; Voll et al., 2003). The reticulate leaf phenotype could be completely restored, when the plants were grown on MS agar plates supplemented with a cocktail of all three AAA, phenylalanine (Phe), tyrosine (Tyr), and tryptophan (Trp) (Streatfield et al., 1999), thus supporting the role of PPT1 in PEP provision for the shikimate pathway. Likewise *cue1* could be complemented by constitutive overexpression of a PPT from cauliflower buds, a PPDK from *Flaveria trinervia* (Voll et al., 2003) or the endogenous plastidial enolase (ENO1, Prabhakar et al., 2010). These enzymes either produce PEP from pyruvate (PPDK) or *via* glycolysis (ENO1) and thus support the idea that PEP limitation in plastids of *cue1* causes metabolic and developmental constraints in the mutant.

The *A. thaliana* genome contains two *PPT* genes (*PPT1* and *PPT2*), which show different temporal and spatial expression profiles (e.g., BAR electronic fluorescence protein [eFP] browser, http://bar.utoronto.ca/efp_arabidopsis/cgi-bin/efpWeb.cgi; Winter et al., 2002). Whilst in leaves *PPT2* (At3g01550) is uniformly expressed, *PPT1* (At5g33320) shows a higher transcript abundance in the mesophyll adjacent to the vascular bundles (Knappe et al., 2003b), whereas in roots *PPT1* transcripts are highly abundant and *PPT2* is only sparsely expressed.

It has been speculated that the developmental constraints in the *cue1* mutant could be based on missing signal molecules deriving, for instance, from phenylpropanoid metabolism, such as neolignans or their glycosides. In tobacco, a similar reticulate leaf phenotype as in *cue1* could be brought about by overexpression of a *MYB* transcription factor from *Anthirrhinum major* (Tamagnone et al., 1998a,b). By competing for their binding sites, the *A. major* MYB factor diminished binding of endogenous MYB factors and thereby suppressed the expression of genes involved in phenylpropanoid metabolism (Tamagnone et al., 1998a,b). The transgenic tobacco plants contained reduced amounts of dehydrodiconiferyl alcohol glucoside (DCG), which had been shown to exert cytokinin-like effects in tobacco callus cultures (Binns et al., 1987; Lynn et al., 1987; Teutonico et al., 1991). However, DCG could not rescue the phenotype in the transgenic tobacco plants when fed via the roots, but it was capable of changing the aberrant shape of autotrophically cultured mesophyll cells generated from the transgenic plants to a wild-type like appearance (Tamagnone et al., 1998b). Thus, in the tobacco system, the occurrence and abundance of DCG seemed to be linked to cell shape and structure of the mesophyll. Hence, it appeared reasonable that *cue1* might be disturbed in a similar metabolic signal (Streatfield et al., 1999; Voll et al., 2003). It has previously been speculated that signal molecules, like DCG, might be produced in roots, where mainly *PPT1* is expressed, and transferred to the shoot via the phloem or xylem, respectively (Voll et al., 2003).

In transgenic tobacco plants, a similar reticulate phenotype has been observed when the cytosolic ENO was repressed by an antisense approach (Voll et al., 2009). In the latter case, PEP generation in the cytosol, i.e., upstream of the PPT, is hampered.

More recently, the role of plastidial PEP for plant development and survival was reinforced by crosses of *cue1* with a mutant defective in ENO1, a single copy gene, expressed in some non-green tissues, for instance in roots, but not in mature leaves (Prabhakar et al., 2009). ENO1 appears to be the only enzyme missing for a complete glycolysis in plastids of leaves and most other tissues. Double mutants defective in both gene functions could not be isolated, indicating that the combined deficiency in PPT1 and ENO1 is lethal to the plants. Indeed, even heterozygous *eno1* mutants in the *cue1* background (abbreviated *cc*E*e*) showed a more severe phenotype than *cue1*. The *cc*E*e* double mutants were severely inhibited in growth, still showed the reticulate leaf phenotype, and produced a high number of aborted seeds, which was based on a maldevelopment of female and male gamethophytes (Prabhakar et al., 2010). In particular the ultrastructure of the pollen was altered in a way that the exine showed aberrant structures suggesting a lower abundance of sporopollonin, which partially derives from phenylpropanoid metabolism. Moreover, a high portion of the pollen could not develop a pollen tube. *PPT1* and *ENO1* are co-expressed during early embryo development (eFP browser, Winter et al., 2002), whereas *PPT2* is expressed right at the beginning and *PPDK* at the end of embryo development. However, neither *PPT2* nor *PPDK* could compensate for the combined deficiency in PPT1 and ENO1. In addition, a high percentage of seeds of the *cc*E*e* plant were smaller in size and contained lowered amounts of lipids (Prabhakar et al., 2010). The latter finding supports the idea that plastidial PEP is not only required for the shikimate pathway, but also, after conversion to pyruvate by pyruvate kinase (PK), for *de novo* synthesis of fatty acids (Ohlrogge and Jaworski, 1997). Furthermore, alterations in the amino acid composition in leaves and flowers of *cue1*, *eno1* and the *cc*E*e* plants pointed at a perturbations in the synthesis of the branched-chain amino acids leucine (Leu) and valine (Val) deriving from plastidial pyruvate (Schulze-Siebert et al., 1984; Singh and Shaner, 1995). Although a sodium/pyruvate co-transporter has recently been identified (Furumoto et al., 2011), pyruvate import into plastids during seed development is unlikely to be the main route of C3 supply. For instance, in plants impaired in plastid-localized PK, seed development is severely compromised (Andre et al., 2007; Baud et al., 2007), indicating that pyruvate is provided from PEP inside the plastids rather than by import from the cytosol. This observation again underlines the importance of plastidial PEP for the survival of plants (Flügge et al., 2011).

In recent years a number of mutants emerged, which share the reticulate leaf phenotype based on lower mesophyll cell density with *cue1*, amongst them the *A. thaliana low cell density1* (*lcd1;* At2g37860) mutant (Barth and Conklin, 2003; González-Bayón et al., 2006), which is allelic to *reticulata* (Rédei and Hirono, 1964). However, the function of the LCD1 protein, which is located in the plastid envelope membrane (*Aramemnon* data base, http://aramemnon.uni-koeln.de/, Schwacke et al., 2004), remains to be elucidated. Further reticulate leaf mutants are the allelic mutants *smo1* and *trp2*, which are defective in the β subunit of tryptophan synthase (Jing et al., 2009), and the *venosa3/6* (*ven3/ven6*) mutants (Mollá-Morales et al., 2011), which show a lesion in carbamoyl phosphate synthase (CPS) involved, for instance, in arginine (Arg) biosynthesis. In contrast to *cue1*, *lcd1*, *smo1*/*trp2*, and *ven3/ven6*, the reticulated *dov1* mutant exhibits chlorotic lesions in the mesophyll at a higher rather than a smaller mesophyll cell density (Kinsman and Pyke, 1998). The mutation of *dov1* has recently been mapped and the defective gene identified as the transaminase catalyzing the first step in purine biosynthesis (Rosar et al., 2012).

The data in this report show that the *cue1* root- and shoot phenotypes are separate phenomena ruling out the involvement of long distance transport of metabolic signals. Moreover, feeding of AAA, DCG, and cytokinins to mature plants could transiently rescue the *cue1* leaf phenotype suggesting a high plasticity in the developmental response of *cue1*. Likewise an inducible RNAi against PPT1 was used to demonstrate the reversibility of the *cue1* phenotype. Most strikingly, the data in this report suggest that PPT1 operates as a PEP importer in most plastids of C3 plants, but might act as an exporter or overflow valve in root plastids.

## Materials and methods

### Plant material, growth conditions and sampling

Seeds of *A. thaliana* ecotype Col-0 as well as the T-DNA insertion mutants *lcd1* (N584529) and *ppdk* (N616157) were obtained from the Nottingham Arabidopsis Stock Centre (NASC). In addition, the lines *cue1-6* (Col-0 background), *cue1-1* and *cue1-3* were used (Streatfield et al., 1999). The line pOCA108 (Bensheim background) served as a control plant for *cue1-1* and *cue1-3* as described in Li et al. (1995). Mutant and wild-type plants overexpressing the PPT from *Brassica oleracea* (var. *cauli*) or the PPDK from *Flaveria trinervia* were described in detail by Voll et al. (2003). Plants were either germinated and grown on soil or on sterile half-strength ½Murashige-Skoog (½MS) agar (horizontally and vertically) in the absence of sucrose for 3–5 weeks. In both cases light/dark cycles of 16/8 h, a day/night temperature of 22/18°C, and a photon flux density (PFD) of 150 μmol·m^−2^·s^−1^ on leaf level were applied in a temperature controlled growth chamber equipped with a mixture of fluorescence tubes (Osram L58W/11-860, L58W/30, L58W/76 and L58W/77). Plant material for RNA extraction was harvested after 5 h in the light. Phytohormones were sterile-filtrated before mixing with the ½MS agar during the cooling process and applied at final concentrations of 1, 10 or 50 μM.

### *A. thaliana* grafts

In order to test the role of long distance transport for the development of the *cue1* phenotype, reciprocal grafts were generated between wild-type and *cue1* mutant lines as described by Christmann et al. (2007).

### Transformation of *A. thaliana*

*Arabidopsis thaliana* was transformed with recombinant *Agrobacterium tumefaciens* by the floral dip method (Bent, 2006). Plants were grown on soil in a temperature controlled greenhouse. In order to increase the number of flowers, the first inflorescences were removed to induce growth of numerous secondary inflorescences. Recombinant *A. tumefaciens* was grown in YEB medium in the presence of the respective antibiotic until an OD_600_ of 0.8–1.0 was approached, concentrated by centrifugation at 2500× g for 15 min, and the sediment re-suspended in infiltration medium supplemented with Silwet L-77 (Lehle Seeds, Round Rock, USA) at a ratio of 1:2000. Before transformation already developed siliques were removed.

### Generation of ethanol-inducible *AtPPT1-RNAi* plants

It was intended to transiently inhibit *PPT1* expression with the aid of an inducible promoter system. The *alcR-alcA* promoter system (Syngenta; Caddick et al., 1998) is based on the dissociation of the *alc* repressor (transcription factor) (*alcR*) from the *alcA* promoter in the presence of ethanol or acetaldehyde. For transient expression, the *PPT1*RNAi constructs is controlled by the *alcA* promoter, whereas the *alcR* transcription factor is driven by the CaMV 35S promoter. For cloning *pGreenII* containing BASTA® resistance was used.

Stable transformed plants were grown for 2–3 weeks on ½MS agar and were then transferred to fresh ½MS agar supplemented with 0.015% (v/v) EtOH. The appearance of the reticulated leaf phenotype was correlated with the abundance of PPT1 transcripts. For withdrawal of EtOH, the transformants were again transferred to fresh ½MS agar plates.

### Amino acid analysis

The analysis of free amino acid contents in *A. thaliana* leaves and roots was essentially carried out as outlined in Prabhakar et al. (2010) by reversed phase HPLC using a Nucleodur 100-5 C18 ec column (Macherey and Nagel). The samples were precolumn derivatised with o-phthalaldehyde (Lindroth and Mopper, 1979).

### Cytokinin and auxin determinations

Rosette leaves and roots of WT and *cue1* plants were harvested at stage 1.03 (12 DAS) from 3 biological replicates. The data on cytokinin and auxin metabolites represent the mean ± SE of six or two measurements for leaves and roots, respectively. Extraction and measurements of auxin and cytokinin levels were performed as described before (Novák et al., 2008, 2012).

### Microscopy and histological analyses

For all histochemical analyses, the Eclipse E800 microscope (Nikon) equipped with differential interference contrast and fluorescence optics was used. Images were captured using a 1-CCD color video camera (KY-F1030; JVC) operated by the DISKUS software package (Technisches Buero Hilgers). For the microscopic analyses of the numbers of sub-epidermal palisade parenchyma cells, leaves were incubated in 0.1% Triton X-100 and centrifuged as described by Horiguchi et al. (2006). Mesophyll cells in an area of 0.05 mm^2^ were counted with the aid of Image J (http://rsb.info.nih.gov/ij/).

### RNA extraction and RT PCR

RNA was extracted according to (Logemann et al., 1987). After treatment with DNA-free DNase (Ambion), oligo(dt)-primed cDNA of total RNA was synthesized using the Bioscript reverse transcriptase (Bioline). PCR using *Taq* polymerase (Promega) according to Mullis et al. (1986) at between 25 and 30 cycles. For the analysis of *PPT1* transcript abundance the following primer combination was used; PPT1for, AGTGCTGAGGAAGGTGATAAC and PPT1rev, AGCCATCAGGGCGAGAGACA. As a loading control *Actin2* was used, with Actin2for, TGTACGCCAGTGGTCCTACAACC and Actin2rev, GAAGCAAGAATGGAACCACCG. Amplified DNA was separated on 0.8–2.0% agarose gels in the presence of Tris-acetate buffer and 5 μg·ml^−1^ ethidium bromide.

### Statistical analysis of experimental data

The data are expressed as mean values ± standard error of the mean (SE) of the indicated number of independent measurements. Significant differences between two data sets were analyzed using the Welch-test, which allows for unequal relative errors between two groups of measurements assuming that a Gauss distribution is applicable (Welch, 1947). Significant differences between more than two data sets were analyzed using single site ANOVA combined with the *post-hoc* Tukey-Kramer test, which allows the comparison of unequal sample sizes and identifies those pairs of values, which are significantly different from each other (Ludbrook, 1998). For data plotting and fitting, SigmaPlot10.0 for Windows (SPSS Inc.) was used.

## Results

### The *cue1* mutants shows at least four separate sub-phenotypes and shares only reticulate leaves with the *lcd1* mutant

**Figure 1:**
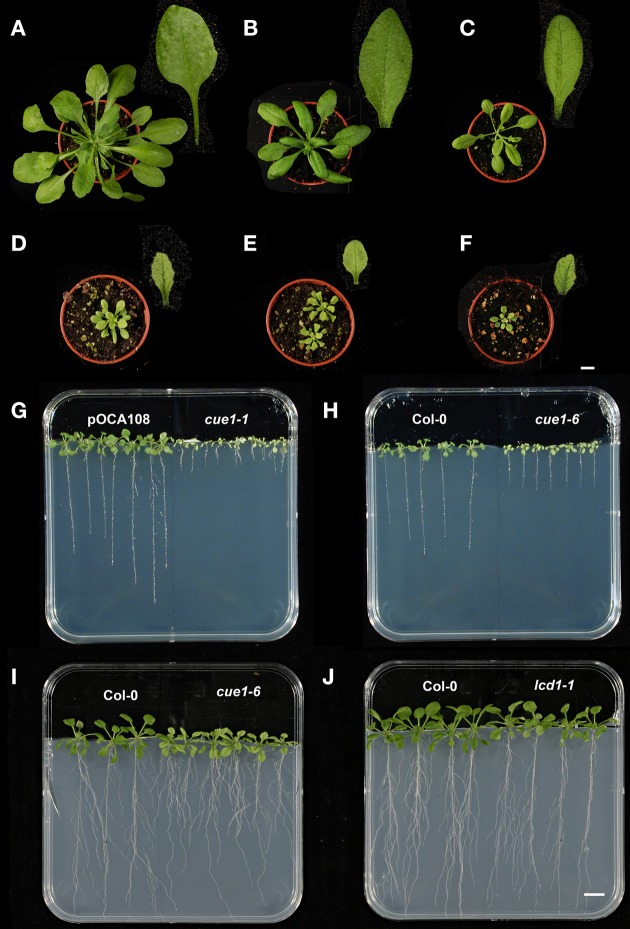
**Shoot and root phenotypes of different *cue1* alleles and *lcd1-1* compared to control plants**. Rosettes and leaves of 30 days old pOCA108 (Bensheim) **(A)**, Col-0 **(B)**, *lcd1-1* **(C)**, *cue1-1* **(D)**, *cue1-1/lcd1-1* double mutants **(E)**, and *cue1-6* **(F)**, as well as root phenotypes of 21 days old pOCA and *cue1-1* **(G)**, Col-0 and *cue1-6* **(H)**, or 35 days old Col-0 and *cue1-6* **(I)**, and Col-0 and *lcd-1* **(J)**. The bars indicate a length of 1 cm referring either to the plants in the pots **(A–F)** or on agar **(G–J)**.

The deficiency in PPT1 results in a complex phenotype of the *cue1* mutant alleles, which can be divided into four separate sub-phenotypes. These are (i) growth retardation of the shoot (Figures 1D,F) compared to the control or wild-type plants (pOCA108, Figure 1A; Col-0, Figure 1B), (ii) a reticulate leaf structure based on smaller mesophyll cells and chloroplasts therein. (iii) More pronounced serrations at the margins of the leaves lead to an altered overall leaf shape (Figure 1D; see Byrne, 2012). Finally (iv) the roots are stunted, most prominently in the *cue1-1* allele (Figure 1G; Bensheim [pOCA] background) compared to *cue1-6* (Figures 1H,I; Col-0 background). In contrast, the *lcd1-1* mutant shares only the reticulate leaf phenotype with *cue1* (Figure 1C). Size of the rosettes, shape of the leaves and root length (Figure 1J) were very similar to the wild type. The *cue1-1* mutant was crossed with *lcd1-1* resulting in the *cue1-1/lcd1-1* double mutant (Figure 1E) that lacked any additional phenotype compared to the *cue1-1* single mutant (Figure 1D).

### Reversed grafting experiments reveal that the root and shoot phenotypes of *cue1* are independent phenomena

**Figure 2:**
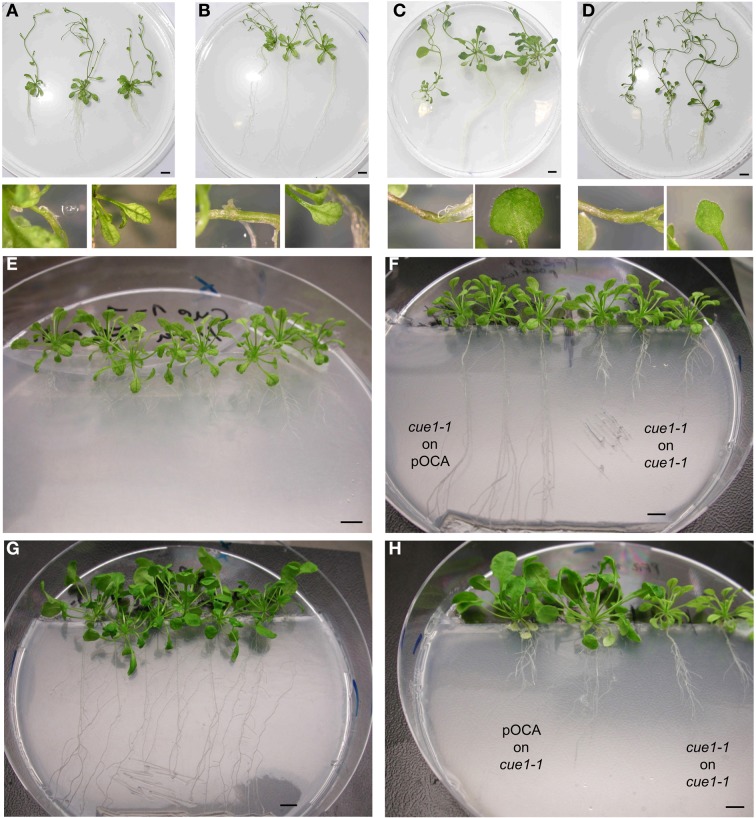
**Development of the *cue1-1* shoot and root phenotypes does not require communication between roots and shoots**. Different scion|root stock combinations of *cue1-1* and pOCA (Bensheim) are shown from two independent grafting experiments. **(A)** *cue1-1*|*cue1-1*(mutant control), **(B)** *cue1-1*|pOCA **(C)**, pOCA|pOCA (wild type control), **(D)** pOCA|*cue1-*. Pictures were taken 35 days after grafting. The small additional pictures show details of the graft union and/or the leaf phenotype. **(E)** *cue1-1* non-grafted **(F)**, *cue1-1*|pOCA grafts compared to *cue1-1*|*cue1-1 grafts* **(G)** pOCA non-grafted, **(H)** pOCA|*cue1-1* grafts compared to *cue1-1*|*cue1-1* grafts. The pictures were taken 18 days after grafting. The bars indicate a length of 1 cm.

In previous reports it was hypothesized that the leaf phenotype of *cue1* results from a deficiency in metabolic signals, which might be produced in the roots, where only *PPT1*, but not *PPT2*, is highly expressed, and transferred to the shoots by long distance transport *via* the phloem or xylem (Knappe et al., 2003b; Voll et al., 2003). We addressed this hypothesis by reversed grafting experiments using young scions or root stocks of *cue1-1* and pOCA, the control plant of *cue1-1* (Figure 2). About 30% of the grafted plants survived and were further analyzed. To our surprise, *cue1-1* scions grafted on pOCA root stocks (*cue1-1*|pOCA plants), still showed smaller, reticulated leaves (Figures 2B,F) and were hence comparable to the controls (Figures 2A,F,H; i.e., *cue1-1*|*cue1-1* plants). Likewise, pOCA roots developed normally despite of the fact that *cue1* scions were grafted on the pOCA root stocks (Figures 2B,F). Moreover, root length and branching was comparable to the control, i.e., pOCA|pOCA plants (Figure 2C). Furthermore, if pOCA scions were grafted on *cue1* root stocks, both shoots and roots retained their phenotypes, i.e., the shoots still looked wild-type like, whereas the *cue1* roots remained stunted in growth (Figures 2D,H). These experiments demonstrate that long distance communication between roots and shoots (and *vice versa*) with regard to the individual phenotypes does not occur. Hence, it is likely that both phenotypes are separate and independent phenomena, based on the deficiency in PPT1 in the individual organs.

### The reticulate leaf phenotype of *cue1* can be reversibly rescued by feeding experiments and an inducible PPT1RNAi approach

**Figure 3:**
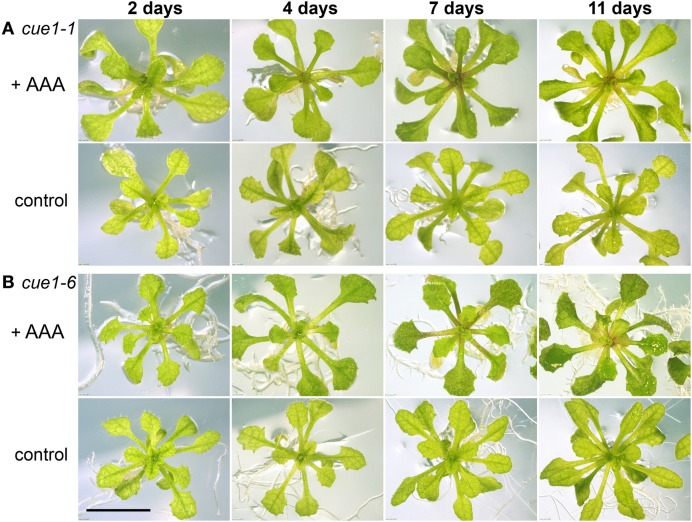
**Rescue of the reticulate leaf phenotype of *cue1***. The alleles of *cue1-1* **(A)** and *cue1-6* **(B)** were fed with a cocktail of AAA. Four week old plants were grown on ½MS agar and then transferred to ½MS agar in the presence or absence (control) of AAA (2 mM each of Phe, Tyr, and Trp). The bar indicates a length of 1 cm.

Previously it has been shown that *cue1* plants grown on a medium supplemented with a cocktail of AAA loose both the growth and reticulate leaf phenotypes. This has been considered as a proof for the essential role of PPT1 in PEP supply for the shikimate pathway in plastids. In order to examine their potential in rescuing the *cue1* phenotype, it was intended to apply a variety of compounds to the *cue1* mutant in a transient system. First it was tested whether mature mutant plants could be transiently rescued by AAA. For this purpose two-week-old plants, with a fully developed leaf phenotype were transferred to ½MS agar supplemented with a cocktail of all three AAA (Figure 3). Indeed, the reticulate leaf phenotype disappeared within seven days of feeding. However, growth of rosette leaves could not be restored within this short time period. Moreover, after back-transfer of the rescued plants to ½MS agar devoid of AAA, the reticulate leaf phenotype re-appeared within three to five days, most prominently in newly developed leaves (not shown).

**Figure 4:**
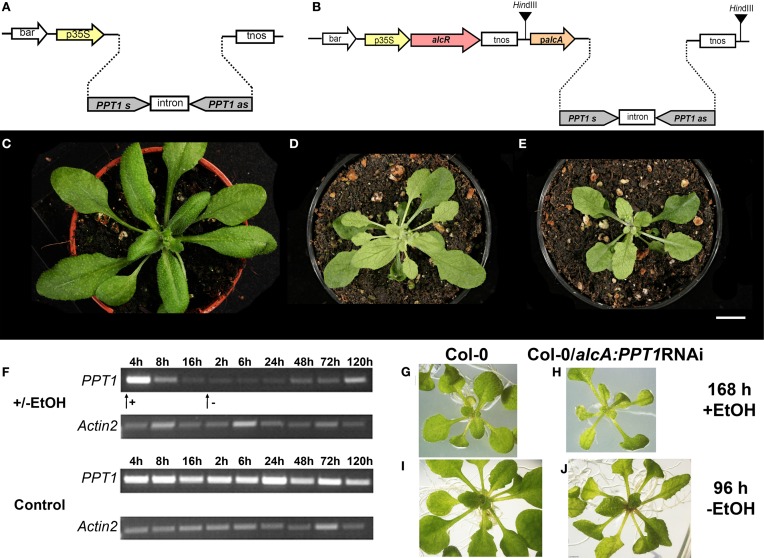
**Repression of *PPT1* expression by a constitutive and inducible RNAi approach**. Constructs are shown for a constitutive *PPT1*RNAi expression driven by the CaMV 35S promoter (p35S) **(A)** or for the EtOH inducible *alcR/alcA* system **(B)**. Phenotypic appearance of Col-0 wild type **(C)**, and two lines expressing the *PPT1RNAi* construct constitutively [**(D)** Col-0/ RNAi(1):*alcA:PPT1*; **(E)** Col-0/RNAi(2):*alcA:PPT1*]. **(F)** Time course of *PPT1* expression (RT-PCR) after induction of *alcA:PPT1*RNAi plants with EtOH compared to a wild-type control. The arrows indicate the addition or withdrawal of 0.015 (v/v)% EtOH. Phenotypic appearance of Col-0 **(G,I)** and Col-0/*alcA:PPT1*RNAi **(H,J)** 168 h after induction with EtOH **(G,H)** or 96 h after withdrawal of EtOH **(I,J)**. The bar in **(C–E)** indicates a length of 1 cm.

In a second approach, *PPT1* expression was transiently repressed using a *PPT1*RNAi construct under the control of the EtOH-inducible *alcR/alcA* promoter (Figure 4B). As a positive control, Col-0 wild-type plants expressing the *PPT1*RNAi construct driven by the constitutive CaMV 35S promoter (Figure 4A) showed all aspects of the *cue1* phenotype (Figures 4D,E) compared to the wild type (Figure 4C). In the absence of the inductor EtOH, Col-0 plants carrying the inducible *PPT1*RNAi construct were indistinguishable from the wild type (not shown). An RT-PCR time series following the induction of the *alcR/alcA- PPT1*RNAi revealed that *PPT1* expression declined severely 8 h after application of with 0.015% EtOH (Figure 4F). The minimum expression level was attained after approximately 16 h. Following the withdrawal of EtOH, the *PPT1* transcript abundance recovered slowly and reached a substantial level after approximately five days. The altered *PPT1* transcript abundance was accompanied by phenotypic changes of the rosette leaves. The reticulate leaf phenotype appeared in the *alcR/alcA-PPT1*RNAi plants within seven days after induction with EtOH (Figure 4H), and disappeared four days after withdrawal of EtOH (Figure 4J) compared to the respective control plants (Figures 4G,I).

Both experiments, the transient application of AAA and the RNAi-dependent suppression or induction of the *PPT1* gene, clearly demonstrate that the *cue1* reticulate leaf phenotype is reversible.

### A neolignan glucoside and the active cytokinin trans-zeatin can rescue the *cue1* phenotype

**Figure 5:**
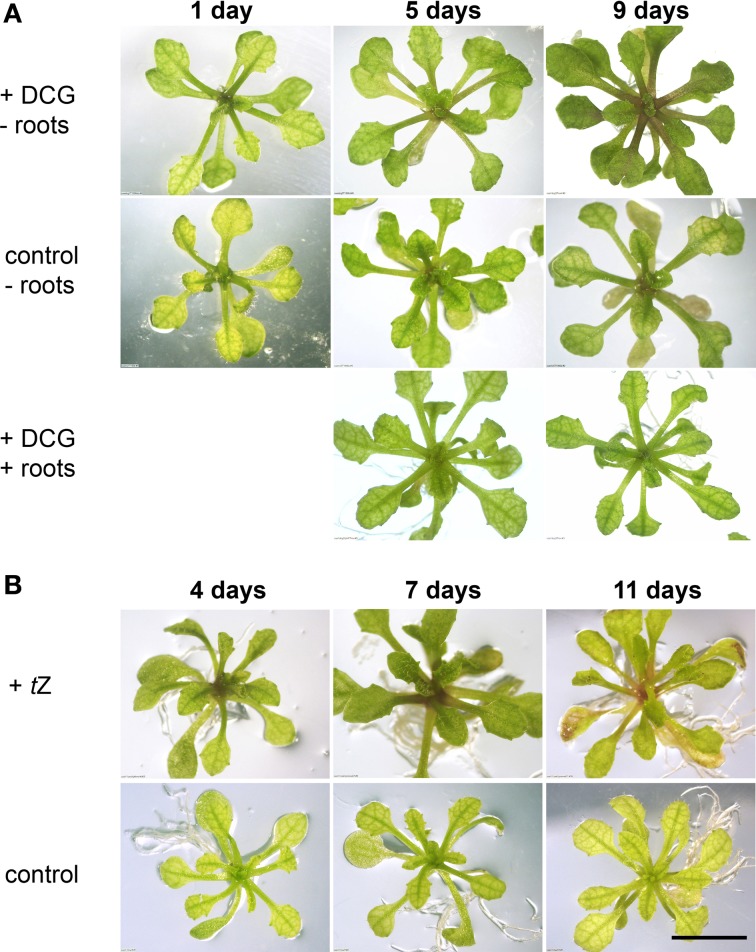
**Feeding of *cue1-1* with DCG (A) or *t*Z (B) compared to an unfed control**. Plants were grown on ½MS agar for four weeks and were then transferred to a medium containing either 20 μM DCG or 1 μM *t*Z. DCG rescued the reticulate leaf phenotype only if the roots were excised (-roots) and the rosettes were placed directly with their cut edges on the agar plates, but not intact plants (+roots). The bar indicates a length of 1 cm.

In previous reports it has been speculated that the *cue1* phenotype might be caused by a depletion of neolignans such as DCA (or its glucoside DCG) deriving from the phenylpropanoid metabolism (Streatfield et al., 1999; Voll et al., 2003). Both the aglycon and glucoside are commercially not available. For our experiments we used DCA and DCG preparations, which were originally synthesized by David Lynn (Orr and Lynn, 1992). Aliquots of the same preparations were also used in the tobacco system by Tamagnone et al. (1998a,b). The *cue1* plants grown on ½MS agar plates were transferred to the same medium supplemented with either 20 μM DCG or DCA, but there was no rescue detectable within one week. However, once the roots had been removed and the *cue1* shoots were placed with their cut surfaces directly on the agar, the reticulate leaf phenotype disappeared within seven to nine days after feeding with DCG (Figure 5A), but not with DCA (not shown). The phenotype re-appeared once new roots had been formed at the cut surface of the shoot. Thus, the newly formed roots blocked the direct uptake of DCG into the plant. After removal of these new roots and back-transfer of the shoots to DCG containing medium, the leaf phenotype disappeared again within about one week (not shown).

This result was quite promising, as it showed that a downstream product of the phenylpropanoid pathway was capable of rescuing the *cue1* phenotype. Feeding of other lignans like lariciresinol und pinoresinol (the aglycons) at a concentration of 20 μM had no rescuing effect on the *cue1* leaf phenotype in the presence or absence of the roots (not shown). In addition, the direct precursor of lignin and lignan biosynthesis, coniferyl alcohol also failed to rescue the reticulate leaf phenotype of *cue1*.

In view of the reported cytokinin-like effects of DCG in the tobacco callus culture system (Binns et al., 1987; Lynn et al., 1987; Teutonico et al., 1991), we applied *trans*-zeatin (*t*Z) to intact *cue1* plants and to *cue1* deprived of the roots. This highly active cytokinin was capable of rescuing the *cue1* phenotype within five to seven days after transfer of the plant to a *t*Z containing medium. Figure 5B shows the effect of *t*Z feeding at a concentration of 1 μM on the reticulated leaf phenotype of *cue1-1*. The phenotype almost completely disappeared after seven days of feeding. However, a prolonged feeding with *t*Z (e.g., for 11 days) led to a general deterioration of the phenotype and bleaching of rosette leaves indicating that cytokinins can have toxic effects when applied at elevated concentrations for a prolonged time period (Hung et al., 2004; Rosar et al., 2012).

Other phytohormones such as the naturally occurring auxin indole acetic acid (IAA), the synthetic auxin naphtyl acetic acid (NAA), abscisic acid (ABA), gibberellic acid (GA3), the ethylene precursor 1-aminocyclopropane-1-carboxylic acid (ACC), salicylic acid (SA), or methyl jasmonate (MeJA) were ineffective in rescuing the leaf phenotypes of *cue1* or *lcd1* (Supplemental Figure 1).

### Both feeding of AAA and *t*Z recovered the mesophyll cell density in *cue1* but not in *lcd1*

**Figure 6:**
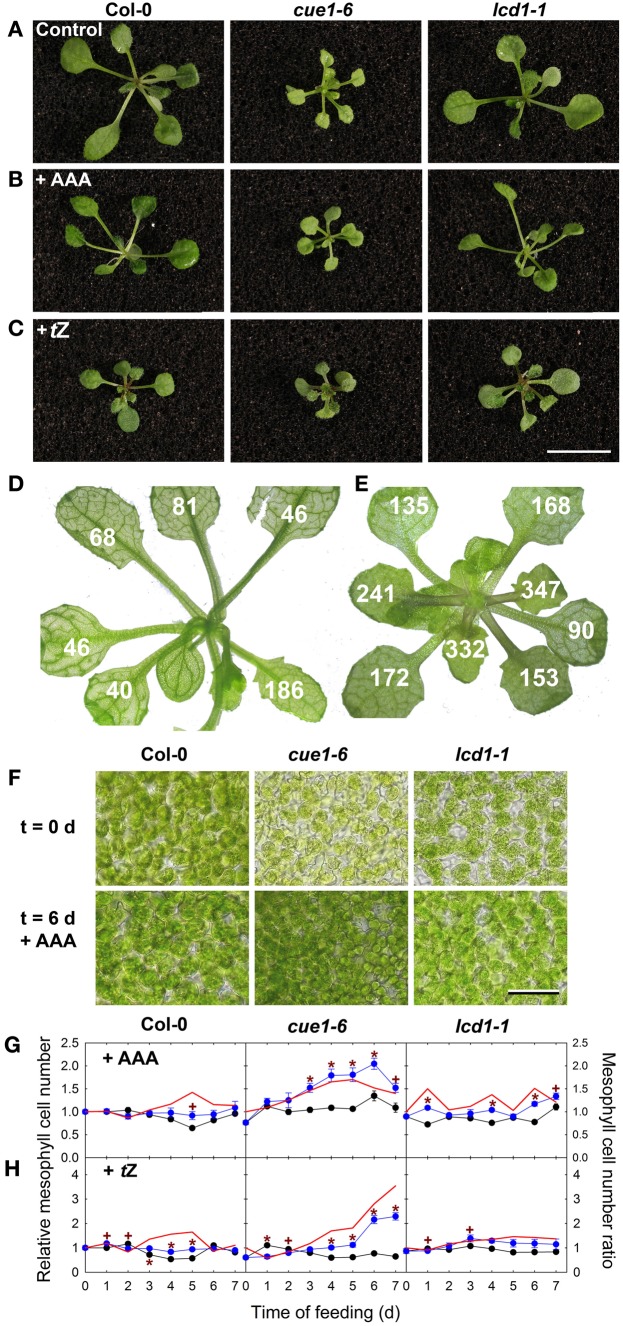
**Effect of AAA and *t*Z on mesophyll cell density in *cue1* and *lcd1* leaves**. Plants were grown for six days on ½MS-agar supplemented with 2 mM each of AAA **(B)** or 1 μM *t*Z **(C)** compared to an unfed control **(A)**. Examples of mesophyll cell density numbers (cells per 0.05 mm^2^) counted in individual leaves of *cue1-6* grown on 1 μM *t*Z **(E)** compared to an unfed control of *cue1-6* **(D)**. Mesophyll cell density of Col-0, *cue1-6* and *lcd1-1* six days after feeding a cocktail of AAA compared to an unfed control **(F)**. Each microscopic picture represents an area of 0.05 mm^2^ with a bar indicating a length of 100 μm. Time course of relative mesophyll cell numbers in leaves of Col-0, *cue1-6*, and *lcd1-1* after feeding of AAA **(G)** or *t*Z **(H)**. The blue or black symbols represent relative mesophyll cell numbers in the presence or absence of the effectors (AAA or *t*Z), and the red line represents the ratio of mesophyll cell numbers ± effector. The initial cell density was about 100, 60, or 86 cells per 0.05 mm^2^ in leaves of Col-0, *cue1-6*, or *lcd1-1*, respectively. The bar in **(C)** represents a length of 1 cm. The data in **(G)** and **(H)** represent the mean ± *SE* of *n* = 10 measurements per time point and condition.

The *A. thaliana lcd1* mutant shares the reticulate leaf phenotype with *cue1*, which is also based on a smaller mesophyll cell size. Although the *lcd1* mutant is not allelic to *cue1* (González-Bayón et al., 2006) and the function of the LCD1 protein is still unknown, we tested whether either a cocktail of AAA or *t*Z were also capable of rescuing *lcd1*. In contrast to previous analyses, which were based on phenotypic comparisons at a macroscopic scale (Figures 6A–C), we investigated the effect of AAA and *t*Z on the level of mesophyll cell density. In *A. thaliana* leaves the mesophyll cell density was highly variable depending on the age or the developmental stage of the leaf (Figure 6D), a fact that had been considered in this study when comparing treated and untreated wild-type or mutant plants. Compared to the wild type the density of the mesophyll cells of *cue1-6* slowly increased within seven days upon feeding of AAA or *tZ* and was almost doubled seven days after onset of feeding (Figure 6). The increase of the mesophyll cell density of *cue1* was related to a macroscopic rescue of the reticulate leaf phenotype (i.e., the leaves appeared uniformly green [Figures 6A–C]). Moreover, the rescue of the mesophyll cell number in *cue1* occurred in a concentration-dependent manner (Supplemental Figures 2, 3). Hence, both substance classes, AAA and cytokinins, were capable of rescuing the underlying cause for the reticulate leaf phenotype in *cue1*, but not in *lcd1*. In the presence of *t*Z, newly emerging leaves of both *cue1* and *lcd1-1* appeared to be greener compared to the control condition (Figures 6A,C), which masked the lack of effect on the cell density in *lcd1-1* (Figure 6H and Supplemental Figures 3K,L).

### Both AAA and *t*Z inhibit root growth in wild-type, *cue1*, and *lcd1* plants

**Figure 7:**
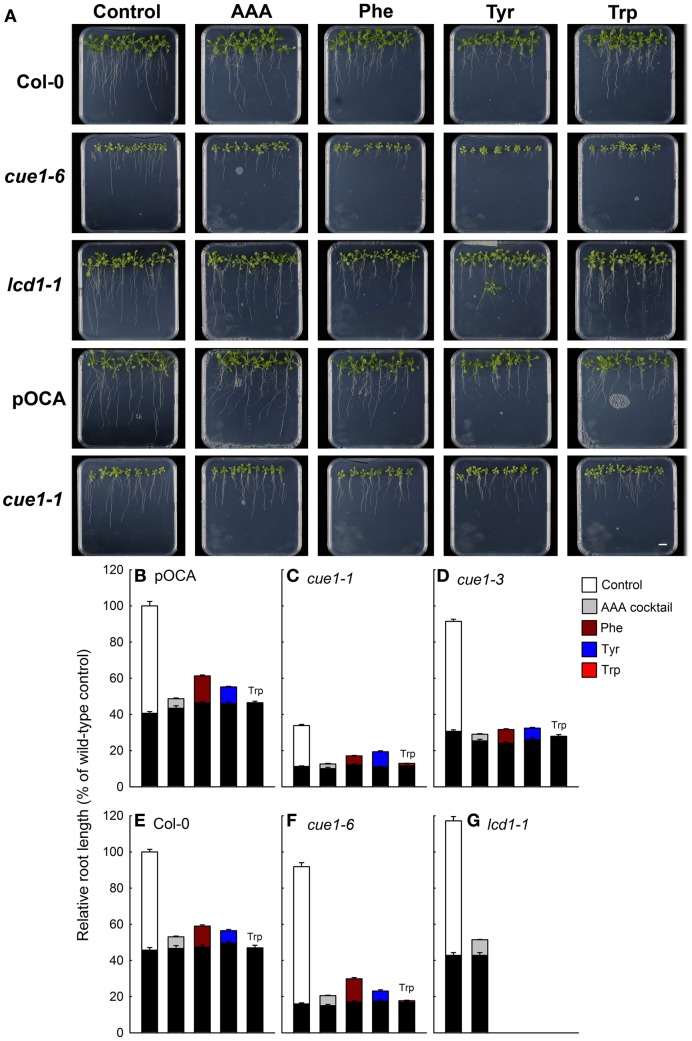
**Root phenotypes seven days after feeding of Col-0, pOCA, *cue1-1*, *cue1-6*, and *lcd1-1* with individual AAA or a cocktail of AAA compared to unfed control plants**. The plants were grown for three weeks on ½MS agar and were then transferred to ½MS agar supplemented with the effectors (2 mM each). **(A)** Phenotypic appearance including root lengths of wild-type/control and mutant plants seven days after start of feeding with AAA (the bar indicates a distance of 1 cm). Relative root lengths of pOCA **(B)**, *cue1-1* **(C)**, *cue1-3* **(D)**, Col-0 **(E)**, *cue1-6* **(F)**, and *lcd1-1* **(G)**. The black bars indicate the relative root lengths before transfer of the plants to AAA containing medium, whereas the stacked white or colored bars refer to the increment growth within seven days. Compared to the control treatment of wild-type and control plants all other relative root lengths were significantly different with *P*-values of at least < 0.01.

Having established that both AAA and *t*Z could rescue the diminished cell density in *cue1*, but not in *lcd1*, we addressed the question as to whether a similar rescue could be brought about for the stunted root phenotype of *cue1*. If, for instance, AAA availability were limiting for growth of *cue1* roots, feeding with a cocktail of AAA should also relief growth retardation of this organ. Strikingly, root growth was further inhibited by AAA application not only in *cue1* but also in wild-type and *lcd1-1* plants (Figure 7A). This inhibitory effect was even more pronounced when Phe, Tyr, and Trp were applied individually. The biosynthesis of amino acids, in particular of AAA, is highly regulated and target of feedback-inhibition by end-products (Tzin and Galili, 2010). Feeding of Trp resulted in a general arrest of root growth in all plant lines (Figures 7B–G). These data show that the application of AAA at millimolar concentrations specifically rescued the leaf, but not the root phenotype of *cue1*. It has been shown earlier that high concentrations of AAA can have an inhibitory effect on plant growth (e.g., Voll et al., 2004). However, even 10-fold lower concentrations of AAA (as well as *t*Z) lacked any promoting effect of the root length of *cue1* (not shown). Hence, a deficiency in AAA as a cause for the stunted root phenotype of *cue1* appears to be unlikely.

Moreover, the effect of individual applications of Phe, Tyr, and Trp on the shoot phenotypes of *cue1* and *lcd1* was also analyzed. In contrast to the AAA cocktail, individual feeding of Phe, Tyr, or Trp (2 mM each) failed to rescue the reticulate leaf phenotype of *cue1* (Supplemental Figure 4).

**Figure 8:**
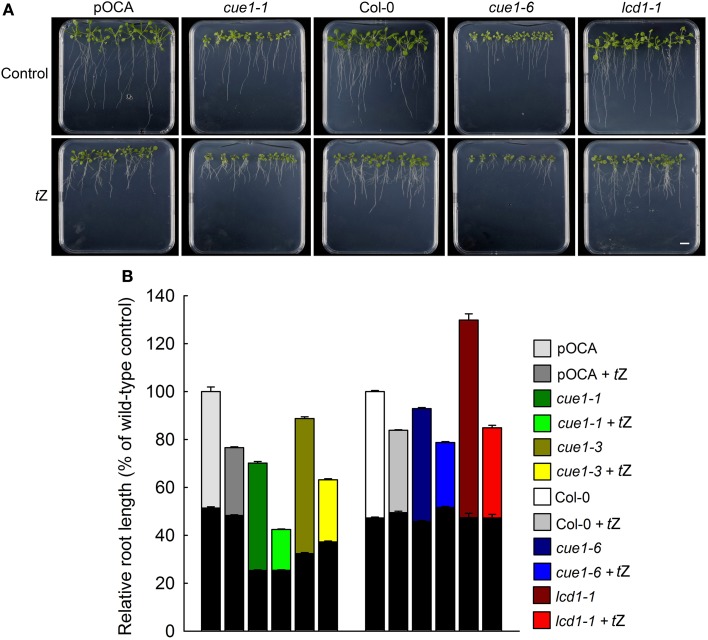
**Root phenotypes seven days after feeding of Col-0, pOCA, *cue1-1*, *cue1-6* and *lcd1-1* with *t*Z**. The plants were grown for three weeks on ½MS agar and were then transferred to ½MS agar supplemented with the effector (1 μM). **(A)** Phenotypic appearance including root lengths of wild-type/control and mutant plants seven days after start of feeding with AAA (the bar indicates a distance of 1 cm). **(B)** Relative root lengths of pOCA, *cue1-1*, *cue1-3*, Col-0, *cue1-6*, and *lcd1-1*. in the absence or presence of *t*Z. The black bars indicate the relative root lengths before transfer of the plants to AAA containing medium, whereas the stacked white or colored bars refer to the increment growth within seven days. Compared to the control treatments all other relative root lengths were significantly different with *P*-values of at least <0.01. The data represent the mean ± *SE* of the mean of *n* = 18 measurements.

It has been shown that active cytokinins, such as benzyladenine (BA), inhibit root-, but promote shoot growth (Cary et al., 1995; Werner et al., 2001, 2003; Howell et al., 2003). Short-term feeding of 1 μM *t*Z led to a substantial inhibition of root growth in all plant lines tested (i.e., pOCA, Col-0, *cue1-1*, *cue1-3*, *cue1-6* and *lcd1-1*, Figure 8). For *cue1-1* the growth inhibition of the roots was even stronger than in the corresponding control plant pOCA. As for the AAA treatment, feeding with *t*Z had opposite effects on the shoot and root phenotype of *cue1*. In Figure 8B, the effect of *t*Z on root growth is also shown for the weak *cue1* allele *cue1-3*.

**Figure 9:**
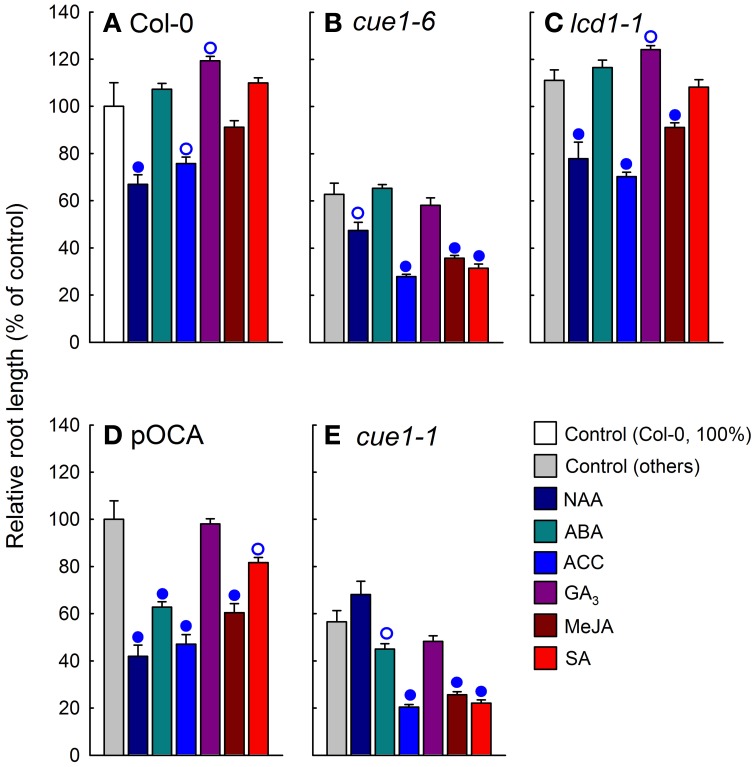
**Effect of phytohormone feeding on root growth of *cue1* and *lcd1* compared to wild-type or control plants**. Individual phytohormones (10 μM each) were fed for seven days to Col-0 **(A)**, *cue1-6* **(B)**, *lcd1-1* **(C)**, pOCA **(D)**, and *cue1-1* **(E)**. The relative root lengths are shown compared to the unfed controls (white bars for Col-0 or light gray bars for the other lines). The circles indicate significant differences in root lengths of fed compared to unfed plants with *P*-values <0.01 (closed circles) and <0.05 (open circles). The data represent the mean ± SE of the mean of *n* = 9 measurements.

As shown in Supplemental Figure 1, phytohormones such as NAA, ABA, GA_3_, ACC (as ethylene precursor), SA or MeJA neither rescued the reticulate leaf phenotype of *cue1* nor of *lcd1*. Here we have studied the effect of phytohormone application on root growth of various *cue1* alleles and *lcd1-1* compared to the respective control plants. In Supplemental Figure 5, the root phenotypes are shown, whereas Figure 9 shows the evaluation of the experiment. The application of NAA at a concentration of 1 μM resulted in an inhibition of root growth in wild-type plants. In *lcd1-1* inhibition of root growth was less pronounced and it was absent in the *cue1-1* and *cue1-6* allele, suggesting that the responsiveness to auxins is diminished in *cue1* roots. The effects of ABA, ACC, GA_3_, and MeJA appeared to be more heterogeneous and responded to the different *A. thaliana* ecotypes rather than to the individual mutations (Figure 9). For instance, ABA promoted root growth in Col-0, but inhibited root growth in pOCA (i.e., Bensheim). Likewise GA_3_ inhibited root growth in *cue1-6*, but had no effect on *cue1-1*. In the presence of SA, root growth of both *cue1* alleles was severely inhibited.

The above data show that a rescue of the stunted root growth could neither be accomplished by the application of AAA nor by phytohormones.

### Do stunted root growth and leaf reticulation of *cue1* respond to plastidial PPDK?

**Figure 10:**
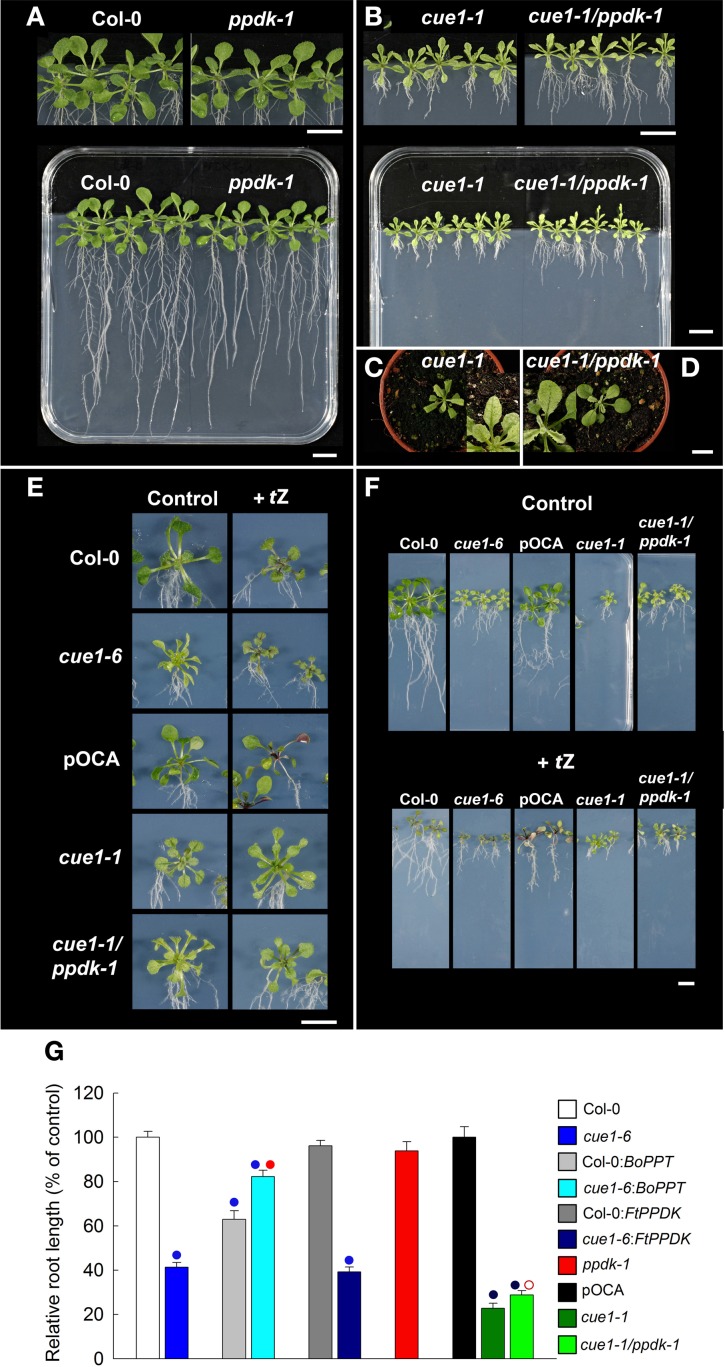
**Effect of *t*Z feeding on the root and leaf phenotypes of *cue1* and *ppdk1-1* single mutants as well as *cue1-1/ppdk* double mutants**. Plants were grown for three weeks vertically on ½MS agar plates or for four weeks on soil **(C,D)**. Root and shoot phenotypes of Col-0 and *ppdk-1* **(A)** as well as *cue1-1* and *cue1-1/ppdk-1* double mutants **(B)**. Col-0, *cue1-6*, pOCA, *cue1-1*, and the *cue1-1/ppdk-1* double mutant fed with 1 μM *t*Z were compared to an unfed control and the leaf **(E)** and root **(F)** phenotypes were analyzed. The data on relative root lengths in **(G)** represent the mean ± SE of *n* = 10–24 replicates. The analyses of root lengths also contain *BoPPT* and *FtPPDK* overexpressing wild-type and *cue1-6* mutant plants. Blue circles indicate significant differences in root length referred to Col-0 (*P* < 0.01), whereas red circles reflect significant changes in root lengths of *BoPPT* overexpressors in the *cue1-6* background compared to the untransformed mutant or of *cue1-1/ppdk1-1* double mutant compared to the *cue1-1* single mutant. Closed or open red circles indicate *P* < 0.01 or *P* < 0.05, respectively. The scale bars in the individual sub-figures indicate a distance of 1 cm.

It has already been shown that constitutive overexpression of a heterologous PPT (from cauliflower buds; *BoPPT*) or of a PPDK from *Flaveria trinervia* (*FtPPDK*) rescued the reticulate leaf phenotype of *cue1* (Voll et al., 2003). In *A. thaliana*, *PPDK* is a single copy gene showing highest expression in pollen and moderate expressed in other cells and tissues. In leaves PPDK is induced during senescence (eFP browser, Winter et al., 2002) and by cytokinin treatment (Brenner et al., 2005). In order to investigate the impact of endogenous PPDK on the phenotype of *cue1*, the *ppdk-1* knockout mutant was isolated, established as homozygous line and crossed to *cue1-1*. The *ppdk-1* single mutant was phenotypically indistinguishable from the wild type (Figure 10A) and the leaves of the double mutant exhibited the *cue1-1* phenotype (Figures 10C,D). However, root growth of the *cue1-1/ppdk-1* double mutant exhibited a slight recovery (Figures 10B,G). Feeding of *t*Z to the *cue1* single mutant and the *cue1-1/ppdk-1* double mutant rescued in both cases the reticulate leaf phenotype (Figure 10E), indicating that the *t*Z effect is independent from *PPDK* induction by cytokinins. Moreover, the additional inhibition of root growth in the presence of *t*Z was identical in *cue1-1* and the *cue1-1/ppdk-1* double mutant (Figure 10F).

Furthermore, root growth was analyzed in the already existing *BoPPT* and *FtPPDK* overexpressing lines (Voll et al., 2003) in the wild-type and *cue1* backgrounds (Figure 10G). The constitutive overexpression of *BoPPT* inhibited root growth in the Col-0 background by 40%, whereas, in the *cue1-6* background, stunted root growth was relieved from about 60% in *cue1-6* to 20% in *cue1-6:BoPPT* compared to the wild type. In contrast, overexpression of *FtPPDK* in the Col-0 background lacked any significant effect on root growth neither in Col-0 nor in *cue1-6*. The roots of the *cue1-6:FtPPDK* line remained stunted, with a length of about 40% of the wild-type. These data show that, apart from the complementation with *BoPPT* only the absence of PPDK in the *cue1-1/ppdk-1* double mutant had a recovering effect on root growth of *cue1*.

### Elevated *t*Z levels in roots of *cue1* are not responsible for stunted root growth

Feeding of *t*Z rescued the reticulate leaf phenotype of *cue1*, but inhibited root growth in wild-type, *cue1*, and *lcd1* plants. Hence, it was investigated whether endogenous levels of cytokinins are altered in both roots and leaves of *cue1* compared to the wild type. The contents of numerous cytokinin and auxin metabolites have been analyzed in roots and leaves (Table 1). As most striking result, there was a significant more than two-fold increase of the active cytokinin *t*Z in roots of *cue1* compared to the wild-type control. The endogenous levels of almost all other isoprenoid cytokinin metabolites were also elevated. This most likely relates to increased cytokinin biosynthesis as the levels of key cytokinin nucleotides (*c/t*ZRMP, iPRMP, DHZRMP) are several times higher in *cue1* plants. Dihydrozeatin (DHZ) and aromatic cytokinins were present at detectable levels.

**Table 1:** **Contents of cytokinin and auxin metabolites in *cue1* rosette leaves and roots**.

**Cytokinins**	**Wild type**	***cue1***	**Wild type**	***cue1***
	**Rosette leaves**		**Root**	
	**pmol·g^−1^ fw**			
*t*Z	0.42 ± 0.01	0.41 ± 0.03	0.37 ± 0.01	0.82 ± 0.05*
*t*ZR	0.73 ± 0.04	1.12 ± 0.03^X^	2.58 ± 0.33	3.96 ± 0.51
*t*Z9G	8.79 ± 0.38	9.24 ± 0.36	14.65 ± 3.82	18.82 ± 5.90
*t*ZOG	3.93 ± 0.20	2.61 ± 0.09**^X^**	5.65 ± 0.06	8.27 ± 0.85
*t*ZROG	0.21 ± 0.01	0.42 ± 0.03**^X^**	0.38 ± 0.02	0.73 ± 0.19
*t*ZRMP	1.25 ± 0.09	2.28 ± 0.31*	0.47 ± 0.10	0.47 ± 0.10
Total *tZ*-types	15.33 ± 0.75	16.08 ± 1.02	24.10 ± 2.78	33.07 ± 5.95
*c*Z	0.10 ± 0.01	0.13 ± 0.01	2.12 ± 0.96	3.64 ± 2.68
*c*ZR	1.11 ± 0.04	2.60 ± 0.29^X^	28.71 ± 16.43	60.01 ± 0.53
*c*Z9G	0.27 ± 0.03	0.45 ± 0.04^X^	0.62 ± 0.30	0.42 ± 0.05
*c*ZOG	0.12 ± 0.02	0.22 ± 0.02**^X^**	1.36 ± 0.52	2.98 ± 2.13
*c*ZROG	0.32 ± 0.02	0.67 ± 0.07**^X^**	6.70 ± 4.33	21.45 ± 9.02
*c*ZRMP	9.48 ± 1.24	26.96 ± 2.82**^X^**	59.66 ± 22.90	139.22 ± 87.60
Total *cZ*-types	11.40 ± 1.15	31.03 ± 2.99	99.17 ± 48.29	227.72 ± 99.77
DHZ	n.d.	n.d.	0.01 ± 0.00	0.04 ± 0.01
DHZR	0.05 ± 0.00	0.06 ± 0.00^+^	0.26 ± 0.13	0.60 ± 0.13
DHZ9G	0.06 ± 0.00	0.06 ± 0.00^+^	0.19 ± 0.07	0.41 ± 0.10
DHZOG	0.38 ± 0.05	0.35 ± 0.03	0.85 ± 0.62	0.77 ± 0.05
DHZROG	0.02 ± 0.00	0.03 ± 0.00	0.05 ± 0.02	0.20 ± 0.07
DHZRMP	0.09 ± 0.01	0.19 ± 0.02**^X^**	0.29 ± 0.10	0.56 ± 0.28
Total DHZ-types	0.60 ± 0.08	0.69 ± 0.07	1.65 ± 0.67	2.58 ± 0.70
iP	0.06 ± 0.01	0.11 ± 0.01^X^	0.14 ± 0.05	0.29 ± 0.08
iPR	0.82 ± 0.05	1.99 ± 0.12^X^	3.02 ± 0.77	7.64 ± 0.70
iP9G	3.15 ± 0.18	6.02 ± 0.15^X^	1.62 ± 0.34	2.49 ± 1.01
iPRMP	1.40 ± 0.08	3.46 ± 0.18^X^	0.25 ± 0.05	0.92 ± 0.44
Total iP-types	5.43 ± 0.46	11.58 ± 0.66	5.03 ± 1.29	11.34 ± 3.55
BAP	0.10 ± 0.01	0.17 ± 0.08	0.09 ± 0.01	0.18 ± 0.13
BAPR	1.32 ± 0.47	0.03 ± 0.00*	0.13 ± 0.00	0.04 ± 0.00
*m*T	0.07 ± 0.00	0.22 ± 0.04**^X^**	0.27 ± 0.00	0.18 ± 0.00
*o*T	0.03 ± 0.01	0.04 ± 0.01^+^	0.03 ± 0.00	0.04 ± 0.00^+^
*o*TR	0.21 ± 0.08	0.13 ± 0.01	0.17 ± 0.00	0.16 ± 0.00
Total Cytokinins	34.49 ± 4.95	59.97 ± 5.68	130.64 ± 34.03	275.31 ± 75.91
**AUXINS**				
IAA	92.47 ± 4.53	107.40 ± 9.07	219.46 ± 14.44	180.18 ± 16.82
IAM	17.08 ± 1.57	16.62 ± 1.20	23.39 ± 3.99	15.49 ± 1.96
IAAGlu	6.29 ± 0.25	7.21 ± 0.32*	9.65 ± 1.27	11.22 ± 1.27

The data on cytokinin metabolites represent the mean ± SE of six or two measurements for shoots and roots, respectively. Only cytokinin metabolites detectable were considered and are abbreviated as follows, tZ, trans-zeatin; cZ, cis-zeatin; t/cZR, t/cZ-riboside; t/cZ9G, t/cZ-9-glucoside; t/cZOG, O-glucosyl-t/cZ; t/cZROG, O-glucosyl-t/cZ-riboside; t/cZRMP, t/cZR-5′-monophosphate; DHZ, dihydrozeatin; DHZR, DHZ-riboside; DHZ9G, DHZ-9-glucoside; DHZOG, O-glucosyl-DHZ; DHZROG, O-glucosyl-DHZ-riboside; DHZRMP, DHZR-5′-monophosphate; iP, isopentenyladenine; iPR, iP-riboside; iP9G, iP-9-glucoside; iPRMP, iPR-5′monophosphate; BAP, 6-benzylaminopurine; BAPR, BAP-riboside; mT, meta-topolin; oT, ortho-topolin; oTR, oT-riboside. The data on auxin metabolites are based on n = 7–8 measurements. The auxin metabolites are indole-3-acetic acid (IAA), indole-3-acetamide (IAM) and the glutamate conjugate of IAA (IAAGlu). Significant differences between the data were calculated according to the Welch test P < 0.05 (labeled with ^*^) or P < 0.01 (labeled with ^X^). Metabolites close to the detection limit are labeled with ^+^.

Despite the fact that neither *t*Z nor *cis*-zeatin (*c*Z) contents showed any significant differences in leaves, there were interesting changes in most less active and inactive cytokinin metabolites, such as ribosides und glucosides (Table 1). Of the 20 glycosides, 13 were significantly increased in leaves of *cue1* compared to the control. The aromatic cytokinin metabolites (BAP, oT, mT) were usually present at detectable levels and did not show any clear trends (Table 1). Strikingly, although the total contents of cytokinin metabolites were almost doubled in leaves and roots of *cue1* compared to the wild type, the amount of active cytokinins was not dramatically changed.

For auxins, there were no pronounced differences in the contents of indole-3-acetic acid (IAA), its glutamate conjugate and the IAA precursor indole-3-acetamide (IAM) (Table 1).

**Figure 11:**
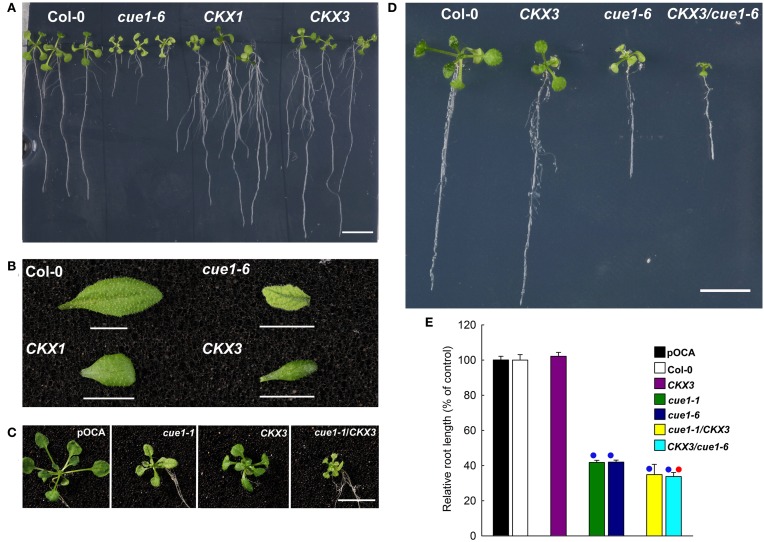
**Root lengths of *cue1* mutants compared to the wild type/control and *CKX* overexpressing lines**. Root **(A)** and leaf phenotypes **(B)** of Col-0, *cue1-6* and *CKX1* and *CKX3* overexpressing lines as well as root **(C)** and leaf phenotypes **(D)** of stable crosses of *cue1-6* with *CKX3* **(C)** or *CKX3* with *cue1-1* **(D)** were assessed three weeks after germination. The scale bars indicate a length of 1 cm. The relative root length in **(E)** is expressed as mean ± SE of *n* = 10–24 replicates. Blue circles indicate significant difference referred to the wild-type or control plants, whereas the red circle refers to a significant difference compared to *cue1-6* (*P*-value < 0.01).

As the amount of *t*Z was almost doubled in roots of *cue1* compared to the wild type, it is conceivable that growth retardation of the roots is a consequence of *t*Z accumulation. It has been shown that the ectopic overexpression of a cytokinin oxidase (*CKX;* CKO) is capable of increasing root growth in transgenic *A. thaliana* (Werner et al., 2003). Two transgenic *A. thaliana* lines overexpressing *CKX* have been crossed with *cue1* and established as stable lines (Figures 11C,D). Roots of the overexpressing lines *CKX1* and *CKX3* (Figure 11A) were longer and more branched than roots from the wild-type or from *cue1-6*, whereas the shoots were retarded in growth (Figure 11B). The rosettes of *CKX3*/*cue1-1* (Figure 11C) and *CKX3*/*cue1-6* (Figure 11D) were also smaller compared to *cue1-1* or *cue1-6*, indicating that active cytokinins are depleted in the shoot due to CKO activity. However, root growth was even further inhibited in *CKX3*/*cue1-6* compared to *cue1-6* (Figure 11D), suggesting that cytokinin accumulation in the roots of the mutant is not the cause for the stunted root phenotype.

### The amino acid composition in roots and leaves suggest different roles of PPT1 in both organs

**Figure 12:**
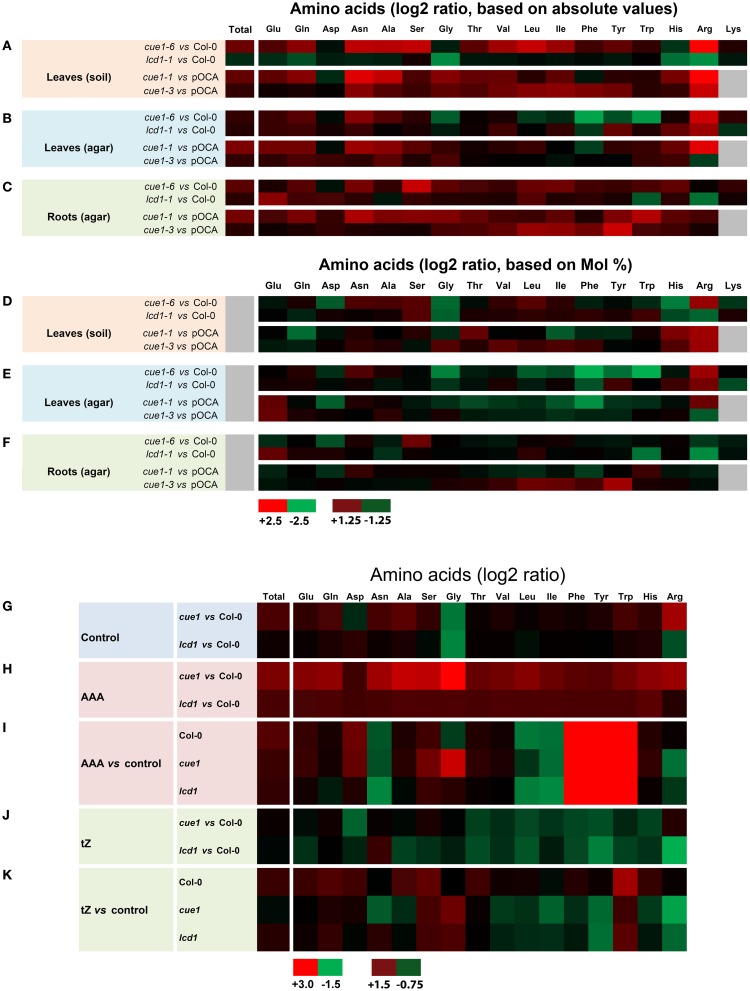
**Heatmap of amino acid spectra in wild-type and mutant plants**. The contents of proteogenic amino acids were determined in leaves of wild-type (Col-0) or control plants (pOCA) as well as *cue1-1*, *cue1-6* and *lcd1-1* mutants grown either on soil **(A,D)** or on ½MS agar **(B,E)**. The amino acid spectra of roots were determined from agar grown plants **(C,F)**. The data are expressed as log2 ratios of absolute **(A–C)** or relative (Mol%) amino acid contents shown in Supplemental Document 1, which also contains a statistical analysis. Amino acid spectra in leaves of wild-type and mutant plants were determined after the plants were fed for seven days either with a cocktail of AAA **(H,J)** or *t*Z **(I,K)** compared to an unfed control **(G)**. For both feeding experiments amino acid ratios were either compared to the wild type **(H,J)** or the unfed control plant **(I,K)**. The maximum (red) and minimum (green) values as well as intermediate values of the color scale for **(A–H)** and **(G–K)** is shown in the figure. Black indicates a value of 0 and in the case of gray shadings no data were available.

In order to further characterize the root- and leaf phenotypes of *cue1*, the spectra of amino acids in both organs of *cue1* as well as in *lcd1* were analyzed (Figures 12A–H, Supplemental Tables 1A–L). There were profound differences in the amino acid composition in rosette leaves depending upon whether the plants were grown on soil (Figures 12A,D) or on ½MS agar (Figures 12B,E). The amino acid spectrum in roots was determined for plants grown on ½MS agar (Figures 12C,F). Statistical ANOVA/*post-hoc* analyses of the amino acid data are contained in Supplemental Table 1.

Strikingly, total contents of proteogenic amino acid in leaves and roots of *cue1* were almost doubled on a fresh weight basis compared to the control plants, irrespectively whether the plants were grown on soil or agar. This increase is mainly based on elevated levels of major amino acids such as Glu, Gln, Asn, Ala, and Ser, whereas Asp or Gly were not appreciably affected. In contrast, *lcd1-1* exhibited similar amino acid levels in leaves and roots as the wild-type with the exception of rosette leaves of agar-grown plants, which contained slightly higher total amino acid levels, based on an increase in Gln und Gly (Supplemental Table 1E). The amino acid spectra were not only dependent on the growth conditions, but also on the ecotype of the plants (i.e., Col-0 for *cue1-6* and *lcd1-1* or Bensheim [pOCA] for *cue1-1* and *cue1-3*). Arg was the only amino acid that showed a significant increase in its absolute and relative contents in leaves of *cue1-1* and *cue1-6*, irrespectively whether the plants were grown on soil or ½MS agar (Figures 12A,B; Supplemental Tables 1A,C,E,G). However, in roots of *cue1-6* or *cue1-1*, Arg contents were not affected and exhibited similar or even lower levels compared to the control plants (Figures 12C,G; Supplemental Tables 1A,C,E,G). All three AAA were significantly decreased in leaves of ½MS agar-grown *cue1-6* (Figures 12B,E), whereas in leaves of soil-grown *cue1-6* AAA were less prominently increased (Figures 12A,D), compared to the other amino acids. The latter also applies to *cue1-1*, with the exception of Phe, which was slightly decreased. The amino acid spectra of soil- and agar-grown *lcd1-1* delivered ambiguous results, as some amino acids such as His and Arg were decreased in soil-grown plants, but increased when the plants were grown on ½MS agar.

There were interesting changes in the amino acid composition in roots compared to leaves of *cue1*, in that contents of AAA or branched-chain amino acids were increased rather than decreased, or remained unaltered compared to the wild type (Figures 12C,F). Moreover, Ser contents were increased in the strong *cue1* alleles *cue1-6* and *cue1-1* (Figure 12C; Supplemental Table 1). In contrast, roots of *lcd1-1* exhibited a decline in Arg and Trp and an increase in the major amino acids Glu, Gln and Asp (Figure 12C, Supplemental Table 1).

### Feeding of *t*Z leads to alterations in the leaf amino acid composition of *cue1* and *lcd1*

In order to gain more information on the metabolic basis for the rescuing effect of AAA and *t*Z on the leaf phenotype of *cue1*, the amino acid composition was determined in leaves of Col-0, *cue1-6* and *lcd1-1*, grown on ½MS agar either in the absence or presence of AAA or *t*Z (Figures 12G–K). In the presence of an AAA cocktail, the content of endogenous AAA was increased substantially 70- to 200-fold in wild-type and mutant plants. The three AAA summed up to 20, 23 and 18 μM·g^−1^ FW in Col-0, *cue1-6* and *lcd1-1* respectively (Supplemental Table 2A) and thus led to an increase in the total amino acid content (Figure 12H; Supplemental Table 2A). Feeding of AAA resulted in a general decrease in Asn, Leu, and Ile, whereas Glu, Asp, and Ser showed a moderate increase in all plant lines (Figure 12I). There was a specific increase of Gly in *cue1-6* and a drop in Arg contents in both *cue1-6* and *lcd1-1* when grown on AAA (Figure 12I). Despite this decrease, the Arg content was still higher in *cue1* compared to the wild type (Figure 12H).

Strikingly, growth on *t*Z resulted in an increase in total amino acid contents in both Col-0 and l*cd1-1*, but a moderate decrease in *cue1-6* (Figure 12K). Moreover, there was a similar pattern in *t*Z dependent changes in the amino acid composition in *cue1-6* and *lcd1-1*. Despite the rescue of the reticulate leaf phenotype in *cue1*, feeding of *t*Z resulted in a decrease of the AAA Phe and Tyr, as well as all three branched-chain amino acids. Furthermore, His and Arg were also decreased in both mutants, whereas Gly and Trp were increased.

## Discussion

In this report we have studied underlying reasons for the shoot- and root phenotypes of the *cue1* mutant based on the loss of function of PPT1. The *lcd1* mutant, which has been analyzed in parallel with *cue1* in some experiments, shares only the reticulate leaf phenotype with *cue1*, but not the growth retardation of the rosettes (compare Figure 1C) or the stunted roots (Figures 1H,J), indicating that impaired mesophyll development seems not to be necessarily coupled with slower rosette or root growth. Thus, the deficiency in PPT1 results in multiple independent phenotypes.

**Figure 13:**
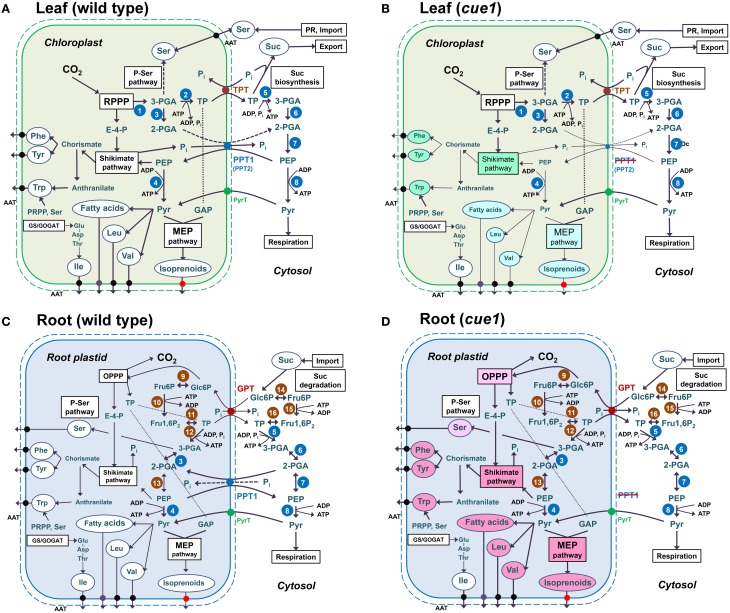
**Anabolic reaction sequences in chloroplasts (A,B) and root plastids (C,D) of wild-type (A,C) and *cue1* mutant plants (B,D)**. In chloroplasts **(A,B)** CO_2_ is assimilated in the reductive pentose phosphate pathway (RPPP; Calvin-Benson cycle) *via* ribulose 1,5-bisphosphate carboxylase/oxygenase (RubisCO;1). Its product, 3-PGA, is then converted to triose phosphates (TP) *via* the subsequent action of phosphoglycerate kinase and NADP glyceraldehydes 3-phosphate dehydrogenase (PGK/NADP-GAPDH; 2). TPs are exported *via* the TPT in counter exchange with inorganic phosphate (P_i_) and subjected to Suc biosynthesis in the cytosol. Suc is the main transport sugar that is exported to the sinks *via* the phloem. In chloroplast glycolysis, 3-PGA can only be metabolized to 2-PGA *via* phosphoglycerate mutase (PGyM; 3), but not further to PEP. Under defined conditions, it can also enter the phospho-serine pathway of Ser biosynthesis. In leaves Ser is, however, mainly produced by photorespiration (PR) or is imported from non-green tissues, such as roots. TPs in the cytosol can undergo the glycolytic conversion to 3-PGA, involving PGK/NAD-GAPDH (5), and further to 2-PGA and PEP by the subsequent action of cytosolic PGyM (6) and enolase (ENO2; 7). PEP has to be imported to the stroma *via* PPT1 or PPT2 in counter exchange with either 2-PGA or P_i_. The latter derive, for instance, from the shikimate pathway, where PEP and erythrose 4-phosphate (E-4-P) serve as precursors. End products of the shikimate pathway are the aromatic amino acids (AAA) Phe and Tyr, which are synthesized from the intermediate chorismate, and Trp, which is synthesized from anthranilate as well as phosphoribosyl pyrophosphate (PRPP) and Ser. AAA are exported *via* amino acid transporters (AAT). PEP can be further metabolized by plastidial (4) or cytosolic (8) pyruvate kinase (PK) yielding pyruvate. Outside the chloroplasts pyruvate is subjected to mitochondrial respiration. In the chloroplast stroma pyruvate can enter *de novo* fatty acid biosynthesis, the production of the branched-chain amino acids Leu and Val as well as the MEP pathway of isoprenoid biosynthesis. The third branched-chain amino acid Ile uses Thr as a precursor. In contrast to wild-type chloroplasts **(A)**, chloroplasts from *cue1* **(B)** suffer from a limitation in PEP provision and processes like the production of AAA *via* the shikimate pathway are impaired (green background) and thus rely on PEP supply by PPT2. Anabolic sequences with pyruvate as precursor would probably be less affected (light blue-green background) as pyruvate might also be supplied by pyruvate transporters (PyrT). In roots **(C,D)** reducing power and metabolic intermediates are provided by the oxidative pentose phosphate pathway (OPPP) with Glc6P as precursor and TP as end products. Cytosolic Glc6P deriving from the degradation of imported sucrose is provided to the plastid by the GPT in counter exchange with either TP or P_i_. Glc6P can be converted to Fru6P by plastidial or cytosolic phosphoglucose isomerise (PGI; 9, 14), activated to Fru1,6P_2_ by phosphofructokinase (PFK; 10, 15), and cleaved by aldolase (11, 16) to TP, which are subsequently converted to 3-PGA, 2-PGA, and PEP by plastidial or cytosolic PGK/NAD-GAPDH (5, 12), PGyM (3, 6), and ENO (7, 13). Note that in root plastids ENO1 is present. The fate of PEP in plastidial and cytosolic metabolism of roots is similar as in leaves. However, a blocked PEP transport across the envelope of root plastids due to a knockout of PPT1 cannot be compensated by PPT2 **(D)**, and would lead to an increased production rather than a depletion of products deriving from PEP and pyruvate (light red background). It is likely that processes like the OPPP or the phospho-serine pathway are also increased by feedback regulatory mechanisms.

The major findings of our investigations can be summarized as follows. (1) Reverse grafting experiments clearly demonstrate that the root- and shoot phenotypes of *cue1* are separate and independent phenomena (see Figure 2), ruling out long distance metabolite signaling as proposed earlier (Streatfield et al., 1999; Knappe et al., 2003b; Voll et al., 2003). (2) The reticulate leaf phenotype is a transient phenomenon that could be brought about or removed by inducible *PPT1*RNAi (see Figure 3) or it could be reversibly rescued by a cocktail of AAA (see Figure 4). (3) The neolignan glucoside, DCG, as a derivative of the phenylpropanoid metabolism downstream of Phe, as well as the active cytokinin *t*Z were capable of rescuing the *cue1* reticulate leaf phenotype (see Figure 5). (4) Feeding of either AAA or *t*Z resulted in an increased cell number in the mesophyll of *cue1*, but not of *lcd1* (see Figure 6 and Supplemental Figures 2,3). (5) AAA or *t*Z inhibited root growth not only in the *cue1* mutant, but also in wild-type and *lcd1* plants (see Figures 7, 8). Feeding of Trp even led to an arrest of root growth in *cue1*. (6) Phytohormones such as auxins, ABA, the ethylene precursor ACC, GA_3_, MeJA and SA had no effect on the leaf phenotype of *cue1*, but affected root growth to various extents (see Figure 9). Roots of *cue1* appeared to be less sensitive towards auxins. (7) Leaves and roots of *cue1* contained higher portions of conjugated, mostly inactive cytokinin metabolites (see Table 1). In roots of *cue1*, the active cytokinin *t*Z was doubled compared to the control, giving rise to the assumption that the stunted root phenotype might be based on elevated *t*Z levels. However, overexpression of *CKX* in the *cue1* background could not rescue the stunted root phenotype (see Figure 11). (8) A careful interpretation of amino acid compositions in leaves and roots of mutant and wild-type plants (see Figure 12) suggested that the role of PPT1 in green and non-green tissues might be different. PPT1 appears to act as a PEP importer in chloroplasts, but as an overflow valve or exporter in root plastids (see Figure 13).

### Local control of organ development vs. long distance signaling

The most surprising finding of the grafting experiments was the lack of any obvious communication between roots and shoots with reference to the individual phenotypes of both organs in *cue1* (Figure 2). If long distance signaling were involved in the aberrant development of the mesophyll or the stunted roots, an at least partial rescue of the individual *cue1* phenotypes would have been expected, depending upon whether *cue1* scions were grafted on wild-type root stocks or *vice versa*. DCG has been proposed earlier as long distance signaling molecule (Voll et al., 2003). Indeed, the application of DCG rescued the reticulate leaf phenotype of *cue1* (due to the lack of availability, the effect of DCG on *lcd1-1* leaves has not been analyzed). However, like in the tobacco mesophyll cell culture system (Tamagnone et al., 1998a,b), DCG appeared to exert only a local effect in *cue1* leaves. It remains to be elucidated whether *A. thaliana* contains detectable amounts of DCG. In contrast to the tobacco system, DCA (the aglycon of DCG) could not rescue the *cue1* phenotype, which suggests that the appropriate glucosyl transferase was either not active or not present in *A. thaliana*. Moreover, like for DCA the non-glycosylated forms of two more lignans, pinoresinol, or lariciresinol, also failed to rescue the reticulate leaf phenotype. More substances ought to be tested to either support or rule out an involvement of lignans in the impaired mesophyll development of *cue1*. It has been shown earlier, that DCG can exert cytokinin-like effects in tobacco callus cultures (Teutonico et al., 1991). Therefore, we tested the effect of cytokinin feeding on the phenotype of *cue1* and *lcd1*.

### The unexpected rescue of the *cue1* reticulate leaf phenotype by *t*Z

Cytokinin metabolism is complex and comprises, besides of highly active compounds like *t*Z, isopentenyladenine (iP) and perhaps cZ, less active or inactive metabolites like ribosides, nucleotides and glucosides (Mok and Mok, 2001). The highly active cytokinin *t*Z was capable of rescuing the reticulate leaf phenotype of *cue1*, but not of *lcd1* (Figure 6). Unlike AAA or DCG, the latter of which eventually derives from Phe as one of the end products of the shikimate pathway, cytokinins are based on purine biosynthesis (Mok and Mok, 2001). Thus, in contrast to AAA, an obvious link of *t*Z to a perturbed plastidial PEP metabolism in *cue1* is missing. Although there are indications for a crosstalk between cytokinins and phenylpropanoids in different plant species, such as tobacco (Gális et al., 2002), maize (Alvarez et al., 2008), or *A. thaliana* (Bhargava et al., 2013), the exact nature of this crosstalk has not yet been resolved. It is, for instance not, clear whether cytokinins act upstream of AAA or their follow-up products (e.g., by inducing biosynthetic reactions of phenylpropanoid metabolism) or downstream of AAA (e.g., by increasing cytokinin biosynthesis or by inhibiting its degradation or conjugation). It is a matter of speculation whether the rescuing effects of both DCG and *t*Z converge further downstream of hormonal signaling and gene regulation.

In contrast to *cue1*, the reticulate phenotype of *lcd1* could neither be rescued by *t*Z nor by AAA. This observation suggests that there are either different developmental bases for this phenotype in *A. thaliana*, or the defect in *lcd1* is located either down- or upstream of the point where AAA and/or cytokinins are able to rescue the leaf phenotype of *cue1*. It has been shown by crossing experiments, that *cue1* is epistatic to *lcd1* (or *re-3*), suggesting that both proteins are involved in the same developmental pathway (González-Bayón et al., 2006). However, a more simplistic view on the rescue of the reticulate leaf phenotype by *t*Z or AAA would be that the cell cycle in the mesophyll of *cue1* is more readily inducible compared to *lcd1* or the wild type. This assumption is supported by the observation that feeding of both AAA and *t*Z significantly increased the cell number in the mesophyll of *cue1* (Figure 6) due to an enhanced mitotic activity (not shown). Enhanced cell numbers have neither been observed in wild-type nor in *lcd1* plants. The hormonal control of the cell cycle in *A. thaliana* comprises an interaction of auxins, cytokinins, ABA and brassinosteroids, which either induce or repress cell cycle regulators (Gutierrez, 2009). It remains to be elucidated whether the regulation of the cell cycle is altered in *cue1*.

### Is there a common denominator for reticulate phenotypes based on lower cell density in the mesophyll?

Bearing the lack of any similarities between *cue1* and *lcd1*, with reference to the rescue by *t*Z, in mind, it was surprising that the pattern of changes in the amino acid composition as a response to *t*Z feeding was very similar in both mutants (Figure 12K). In particular branched-chain amino acids, as well as Phe, Tyr, His, and Arg were decreased, whereas Trp and Gly were increased. In particular Arg contents in leaves of *cue1* were consistently elevated in our study (Supplemental Tables 1, 2) and in earlier reports (Streatfield et al., 1999), and are, hence, back to wild-type level by feeding of AAA or *t*Z. Increased Arg contents have been proposed to be involved in nitric oxide (NO) production in the *nox1* mutant, which is allelic to *cue1* (He et al., 2004). NO is an important signaling molecule in plants and can, for instance, interfere with phenylpropanoid metabolism and thereby regulate responses to pathogen attacks (Zeier et al., 2004). The synthesis of NO was proposed to be either catalyzed by NO synthase (NOS) *via* Arg and citrulline or by a side reaction of nitrate reductase *via* nitrite (Wendehenne et al., 2001; del Rio et al., 2004). A loss of function mutant of NOS (*nos1*) contained lower NO contents in roots (Guo et al., 2003). In another system, feeding of the NO donor sodium nitroprusside resulted in a further inhibition of root growth in *nox1* (*cue1*), but not in the wild type (He et al., 2004). More recent findings suggest that the NOA (NITITE OXIDE ASSOCIATED PROTEIN1)/RIF1 (RESISTANT TO INHIBITION BY FOSMIDOMYCIN) protein, a plastidial GTPase, is involved in the regulation of NO synthesis (Gas et al., 2009).

In contrast to *cue1*, the *ven3/ven6* mutants defective in CPS and, hence, Arg biosynthesis show lower Arg levels (Mollá-Morales et al., 2011). Thus modified Arg levels are not *per se* responsible for the reticulate leaf phenotype. In line with the defect in *cue1*, the reticulate *trp2* mutant, impaired in Trp biosynthesis, is deficient in the synthesis of AAA. Further comparative analyses are required to reveal common mechanistic bases for the developmental constraints in the mesophyll. A detailed update on reticulate mutants is contained in Lundquist et al. (2013). Furthermore, these authors discuss a “supply” or “signaling” hypothesis (see also Rosar et al., 2012), which are both based on the assumption that the bundle sheath of C3 plants plays a profound role in mesophyll development. Although bundle sheath cells in C3 plants are morphologically similar to mesophyll cells, they can conduct a distinct metabolism (Hibberd and Quick, 2002), or their chloroplast carry transporters, like for instance GPT1 (Kunz et al., 2010) or PPT1 (Knappe et al., 2003b), which are not or only weakly expressed in mesophyll cells.

### Root growth is inhibited in the absence of PPT1

Although AAA and *t*Z were capable of rescuing the reticulate leaf phenotype, they further deteriorated the stunted root phenotype of *cue1* and also inhibited root growth in wild-type and control plants (see Figures 7, 8). Although the endogenous *t*Z contents in *cue1* roots were more than doubled compared to wild-type or control roots, it is less likely that elevated cytokinin levels contribute to the stunted root phenotype. For instance, overexpression of the cytokinin-degrading enzyme CKO3 in the *cue1* background could not rescue the root phenotype of *cue1*. In contrast, root growth was even further inhibited in the overexpressing lines (Figure 11E). It has been shown earlier that overexpression of *CKX1* and *CKX3*, which are localized in the vacuole, exerted the strongest cytokinin dependent deficiency syndrome, such as promotion of root growth (Werner et al., 2003).

The most pronounced inhibitory effect on root elongation by individual AAA was observed after feeding of Trp, which resulted in an arrest of root growth. This might be due to feedback inhibition of the initial step of the shikimate pathway by Trp (e.g., Herrmann and Weaver, 1999) and hence a depletion in Phe and Tyr in the presence of Trp. Moreover, Trp is also the precursor for auxin biosynthesis (e.g., Ljung, 2013) and thus a link between amino acid metabolism and hormonal signaling. The observation that *cue1* was less responsive to the inhibitory effect of NAA on root growth might point into the direction of hormonal signaling. Although endogenous auxin levels in roots of *cue1* were not different from wild-type or control plants (Table 1), an altered sensitivity toward auxin might contribute to the stunted root phenotype, an observation that deserves more attention in future experiments. Cytokinins can interfere with the expression of auxin related genes both in roots and shoots (Brenner and Schmülling, 2012). Probably increased endogenous *t*Z levels in roots of *cue1* are involved in the decreased auxin sensitivity. Endogenous levels of Trp were more steeply increased in roots of the *cue1-1* allele compared to *cue1-6* or the weak *cue1-3* allele. Thus, Trp accumulation in the roots correlated to some extend with the inhibition of root growth in the individual *cue1* mutant alleles (compare Figures 8B, 12C). Moreover, *lcd1*, which contained lower levels of Trp, developed longer roots (Figures 8B, 12C). Again, any direct link between Trp contents, sensitivity toward auxin, and root length in *cue1* or *lcd1* remains to be established.

Plants with an impaired phospho-Ser (P-Ser) pathway of *de novo* Ser biosynthesis also display a stunted root phenotype (Benstein et al., 2013). Due to a lesion in the initial step of this pathway, i.e., in phosphoglycerate dehydrogenase (PGDH), particularly in PGDH1, Ser biosynthesis in roots and other non-photosynthetic tissues is hampered. As Ser is also required for Trp biosynthesis, the stunted root phenotype of PGDH1 knockdown plants probably reflects diminished Trp and concomitantly auxin levels in the roots. On a whole seedling scale, IAA levels were clearly diminished in the knockdown plants compared to the wild type (Benstein et al., 2013). However, in the case of the PGDH1 knockdown lines, the demand for Trp seems not to be the only reason for the root growth defects, as feeding with Trp did not rescue the phenotype.

### The role of PPT1 in root plastids

The pleiotropic phenotypes of the *cue1* mutant, which are manifested independently in different organs, such as leaves and roots, point at the necessity to re-evaluate the putative roles of the PPT in plastids of autotrophic and heterotrophic tissues. It is well established and supported by experimental data that most plastids, particularly chloroplasts, rely on the import of PEP not only for the shikimate pathway, but also for plastidial pyruvate-derived pathways like *de novo* fatty acid biosynthesis, the synthesis of Val and Leu and the MEP pathway. Strikingly, homozygous double mutant with a combined loss of function in PPT1 and ENO1 were lethal (Prabhakar et al., 2010). Likewise, the inability to produce pyruvate from PEP in PK deficient plants (Andre et al., 2007; Baud et al., 2007) resulted in diminished oil contents of the seeds. Unlike mixotrophic plastids, i.e., those in embryos during the early stages of seed development, which are able to provide PEP both by a complete plastidial glycolysis and by import from the cytosol, chloroplasts are entirely dependent on PEP import *via* the PPT, because chloroplasts lack ENO1 (Prabhakar et al., 2009). In contrast, *ENO1* expression in roots is appreciably high and thus can support PEP provision by plastidial glycolysis. Hence, PEP import into root plastids by a PPT might not be necessary. Moreover, PEP accumulation inside the plastids due to a lack in the PPT as PEP exporter might even be harmful to the plants and results in stunted roots. There are several lines of evidence that support this idea. (1) If a depletion in products of the shikimate pathways deriving from plastidial PEP were the major cause for the stunted root phenotype of *cue1*, wild-type scions grafted on *cue1* root stocks would eventually rescue the phenotype as, for instance, aromatic amino acid as well as branched-chain amino acids can be transported *via* the phloem (Winter et al., 1992). (2) The absolute contents of AAA as well as of the branched-chain amino acid Leu are increased in *cue1* roots as PEP can be generated *via* glycolysis, e.g., from imported Glc6P (Kunz et al., 2010). (3) Ser as product of the P-Ser pathway (Benstein et al., 2013; Cascales-Miñana et al., 2013) is increased in *cue1* roots, probably because products of plastidial glycolysis downstream of 3-PGA, the substrate of the P-Ser pathway, accumulate. (4) Increased Ser contents correlate with increased levels of Trp, which has been shown to be toxic to root growth at high concentration. (5) The prenyl side chain of the cytokinin *t*Z, which is substantially increased in *cue1* roots, derives from the plastidial MEP pathway (Kasahara et al., 2004).

Figure 13 shows the proposed consequences a deficiency in the PPT1 has on leaf and root metabolism. In chloroplasts, the lack of PPT1 can be partially compensated by PPT2 (Figure 13B), which is highly expressed in leaves, but not in roots. The analysis of double mutants defective in both PPT1 and PPT2 might shed further light on the organ specific and developmental roles of both transporters. As the PPT acts as an importer in chloroplasts (Figure 13A), products deriving from either plastidial PEP, such as AAA, or pyruvate, like fatty acids, branched-chain amino acids and products of the MEP pathway are diminished (indicated by the green background color). However, it has been shown recently that the MEP pathway is hampered in plants with an impaired Na-pyruvate transporter (Furumoto et al., 2011) suggesting that pyruvate as one of the substrates of the MEP pathway can be imported directly from the cytosol. Hence, products deriving from plastidial pyruvate are shown with a light green background color (Figure 13B). Due to the presence of ENO1 in root plastids (Figure 13C), PEP import from the cytosol is not required. Moreover, in contrast to chloroplasts, a knockout of PPT1 cannot be compensated by PPT2 (Figure 13D). We propose that the PPT in root plastids acts as an overflow valve for PEP and/or 2-PGA and thus connects plastidial with cytosolic glycolysis. In the absence of the PPT, products deriving from PEP and pyruvate metabolism accumulate (indicated by a red background colors). Key enzymes of the MEP pathway, 1-deoxy-D-xylulose 5-phosphate reductoisomerase or 1-deoxyxylulose 5-phosphate synthase show a substantial expression in roots (Estévez et al., 2000; Carretero-Paulet et al., 2002; eFP browser, Winter et al., 2002). Likewise, fatty acid biosynthesis and the production of Val and Leu are increased. The branched-chain amino acid Ile is probably less affected, as it is synthesized from Thr and eventually from Glu, which in root plastids is provided by NADH-dependent glutamate synthase. The redox equivalents required for this reaction are provided by a plastidial NAD malate dehyrogenase (Selinski et al., 2014).

## Conclusions and outlook

The data presented here combined with recent publications (Prabhakar et al., 2009, 2010) suggest that the leaf and root phenotypes of *cue1* are based on different roles of the PPT in both organs, i.e., a PEP importer in leaf chloroplasts as well as in mixotrophic plastids of developing seeds, but an overflow valve in root plastids. Most likely, the accumulation of Trp or other AAA in roots of *cue1* combined with a reduced sensitivity toward auxins might form the underlying reason for the stunted root phenotype of *cue1*. This view is re-enforced by a partial rescue of the stunted root phenotype in *cue1* plants overexpressing the PPT from cauliflower buds. Moreover, an increase in plastidial PEP production by constitutive overexpression of a PPDK from *F. trinervia*, rescued the leaf, but not the stunted root phenotype, whereas the additional knockout of the *A. thaliana* PPDK in the *cue1* background had a small recovering effect on root growth. However, the rescue of the root phenotype by overexpressing ENO1 from *A. thaliana* in the *cue1* background (Prabhakar et al., 2010) would only fit into our model, if co-suppression of ENO1 in the roots were assumed, which would lead to a diminished PEP production and hence a relief in the accumulation of products deriving from PEP in root plastids. The respective lines ought to be re-evaluated against this background.

It is surprising that *cue1* leaves accumulate substantial amounts of Arg, whereas an inhibition of CPS in the *ven3/ven6* mutants (Mollá-Morales et al., 2011) results in diminished Arg and citrulline contents, despite of an identical leaf phenotype. Metabolome combined with transcriptome analyses of *cue1* as well as *lcd1* are on the way and will shed more light on the metabolic basis for lowered cell densities in the leaves of both mutants.

### Conflict of interest statement

The authors declare that the research was conducted in the absence of any commercial or financial relationships that could be construed as a potential conflict of interest.

## Supplementary material

Data Sheet 1 contains two Supplemental Tables (Tables 1A–L; Tables 2A–D). Table 1 comprises absolute or relative amino acid contents in leaves or roots of *cue1* and *lcd1* mutant compared to wild-type or control plants grown either on soil or on ½MS agar (for determinations in roots) as well as statistical analyses (ANOVA/Tukey Kramer) for each experiment. Table 2 contains data on the amino acid composition in *cue1*, *lcd1* and Col-0 after feeding of AAA or *t*Z compared to unfed control plants as well as statistical analyses (ANOVA/Tukey Kramer). Furthermore Data Sheet 1 contains five Supplemental Figures.
