# Supplementary material for: Reticulate leaves and stunted roots are independent phenotypes pointing at opposite roles of the phosphoenolpyruvate/phosphate translocator defective in cue1 in the plastids of both organs
Source: Front Plant Sci. 2014 Apr 8;5:126. doi: 10.3389/fpls.2014.00126 (PMC3986533; doi:10.3389/fpls.2014.00126)
Supplement: Supplementary file 1 [file DataSheet1.PDF]

Supplemental Document 1, Table 1 A. Amino acid contents in rosette leaves of soil-grown plants.

Amino acids	Col-0 (A)	*cue1-6* (B)	*lcd1-1* (C)	pOCA (D)	*cue1-1* (E)	*cue1-3* (F)
				(μmol·g^{-1} fw)		
**Glu**	2.533 ± 0.352	4.397 ± 0.198	1.647 ± 0.186	2.095 ± 0.086	3.971 ± 0.253	2.333 ± 0.082
**Gln**	2.665 ± 0.647	6.763 ± 0.485	1.273 ± 0.365	1.491 ± 0.109	3.739 ± 0.601	1.775 ± 0.123
**Asp**	2.214 ± 0.390	1.898 ± 0.160	1.681 ± 0.220	2.245 ± 0.147	1.512 ± 0.072	2.336 ± 0.101
**Asn**	0.525 ± 0.096	1.879 ± 0.074	0.377 ± 0.084	0.431 ± 0.036	1.961 ± 0.116	1.050 ± 0.087
**Ala**	0.824 ± 0.131	2.861 ± 0.229	0.624 ± 0.098	0.582 ± 0.037	2.196 ± 0.125	1.037 ± 0.053
**Ser**	0.839 ± 0.117	3.156 ± 0.235	0.984 ± 0.093	1.450 ± 0.091	2.392 ± 0.174	2.192 ± 0.273
**Gly**	0.762 ± 0.072	0.572 ± 0.051	0.196 ± 0.034	0.119 ± 0.009	0.291 ± 0.086	0.226 ± 0.087
**Thr**	0.640 ± 0.109	1.191 ± 0.094	0.515 ± 0.074	0.547 ± 0.032	1.064 ± 0.066	0.951 ± 0.068
**Val**	0.092 ± 0.016	0.272 ± 0.022	0.076 ± 0.010	0.131 ± 0.009	0.261 ± 0.025	0.274 ± 0.018
**Leu**	0.021 ± 0.004	0.081 ± 0.007	0.019 ± 0.003	0.086 ± 0.007	0.112 ± 0.025	0.210 ± 0.012
**Ile**	0.036 ± 0.007	0.102 ± 0.007	0.031 ± 0.006	0.066 ± 0.004	0.124 ± 0.022	0.169 ± 0.011
**Phe**	0.020 ± 0.003	0.030 ± 0.002	0.018 ± 0.003	0.070 ± 0.007	0.057 ± 0.013	0.163 ± 0.015
**Tyr**	0.021 ± 0.002	0.041 ± 0.002	0.017 ± 0.001	0.038 ± 0.002	0.051 ± 0.009	0.080 ± 0.013
**Trp**	0.041 ± 0.001	0.058 ± 0.002	0.041 ± 0.002	0.043 ± 0.003	0.057 ± 0.005	0.058 ± 0.005
**His**	0.039 ± 0.006	0.023 ± 0.004	0.013 ± 0.003	0.007 ± 0.000	0.016 ± 0.001	0.010 ± 0.001
**Arg**	0.113 ± 0.021	0.658 ± 0.047	0.028 ± 0.004	0.115 ± 0.013	0.652 ± 0.085	0.448 ± 0.068
**Lys**	0.158 ± 0.015	0.199 ± 0.016	0.127 ± 0.009	n.d.	n.d.	n.d.
**Total**	**11.5 ± 2.00**	**24.2 ± 1.6**	**7.7 ± 1.2**	**9.5 ± 0.6**	**18.5 ± 1.7**	**13.3 ± 1.0**

The data represent the mean ± SE of n = 5 replicates.

Supplemental Document 1, Table 1 B. Statistical analysis (ANOVA/Tukey-Kramer) of absolute amino acid contents in rosette leaves of soil-grown plants shown in Table 1 A.

Three comparisons: **Six comparisons:**

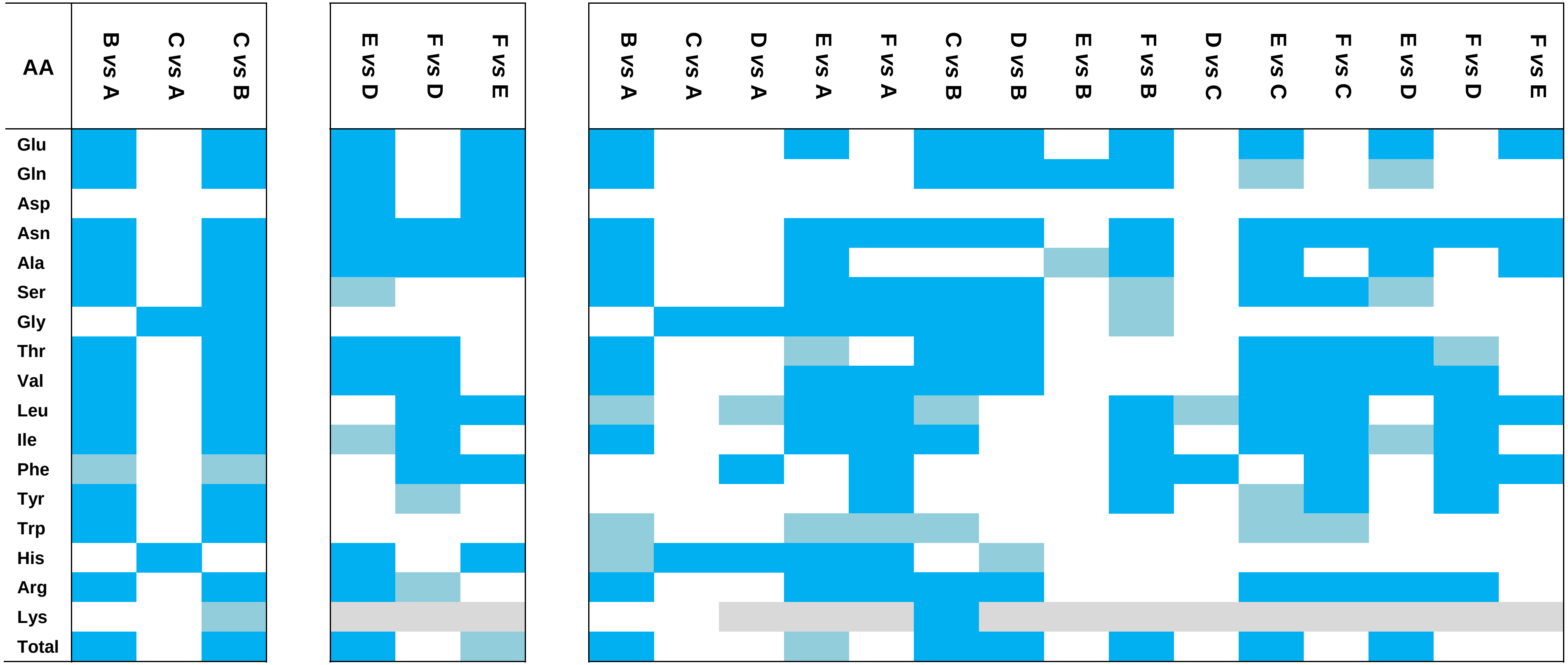

Supplemental Document 1, Table 1 C. Relative amino acid contents in rosette leaves of soil-grown plants calculated from Table 1 A.

**Amino acids**	**Col-0 (A)**	***cue1-6* (B)**	***lcd1-1* (C)**	**pOCA (D)**	***cue1-1* (E)**	***cue1-3* (F)**
				**(%)**		
**Glu**	21.95 ± 3.05	18.18 ± 0.82	21.49 ± 2.42	22.02 ± 0.91	21.52 ± 1.37	17.52 ± 0.62
**Gln**	23.09 ± 5.60	27.97 ± 2.00	16.61 ± 4.75	23.60 ± 1.55	8.19 ± 0.39	17.55 ± 0.76
**Asp**	19.18 ± 3.38	7.85 ± 0.66	21.92 ± 2.87	15.24 ± 0.96	12.96 ± 0.94	16.46 ± 2.05
**Asn**	4.55 ± 0.83	7.77 ± 0.31	4.91 ± 1.09	1.25 ± 0.10	1.58 ± 0.46	1.70 ± 0.65
**Ala**	7.14 ± 1.14	11.83 ± 0.95	8.14 ± 1.28	5.75 ± 0.34	5.77 ± 0.36	7.14 ± 0.51
**Ser**	7.27 ± 1.02	13.05 ± 0.97	12.84 ± 1.21	15.68 ± 1.14	20.26 ± 3.26	13.33 ± 0.92
**Gly**	6.61 ± 0.62	2.37 ± 0.21	2.55 ± 0.44	0.90 ± 0.07	0.61 ± 0.14	1.58 ± 0.09
**Thr**	5.54 ± 0.94	4.93 ± 0.39	6.72 ± 0.97	6.12 ± 0.39	11.90 ± 0.68	7.79 ± 0.40
**Val**	0.79 ± 0.14	1.12 ± 0.09	0.99 ± 0.13	0.69 ± 0.05	0.67 ± 0.12	1.27 ± 0.08
**Leu**	0.18 ± 0.03	0.33 ± 0.03	0.25 ± 0.04	1.37 ± 0.09	1.42 ± 0.14	2.06 ± 0.13
**Ile**	0.31 ± 0.06	0.42 ± 0.03	0.40 ± 0.07	0.74 ± 0.07	0.31 ± 0.07	1.23 ± 0.11
**Phe**	0.17 ± 0.03	0.13 ± 0.01	0.23 ± 0.04	0.40 ± 0.02	0.27 ± 0.05	0.60 ± 0.10
**Tyr**	0.18 ± 0.02	0.17 ± 0.01	0.22 ± 0.02	0.46 ± 0.03	0.31 ± 0.03	0.43 ± 0.04
**Trp**	0.35 ± 0.01	0.24 ± 0.01	0.54 ± 0.03	0.08 ± 0.00	0.09 ± 0.00	0.08 ± 0.01
**His**	0.33 ± 0.05	0.10 ± 0.02	0.17 ± 0.04	4.53 ± 0.37	10.62 ± 0.63	7.89 ± 0.65
**Arg**	0.98 ± 0.19	2.72 ± 0.19	0.37 ± 0.05	1.20 ± 0.14	3.53 ± 0.46	3.36 ± 0.51
**Lys**	1.37 ± 0.13	0.82 ± 0.06	1.65 ± 0.12	n.d.	n.d.	n.d.

The data represent the mean ± SE of n = 5 replicates.

Supplemental Document 1, Table 1 D Statistical analysis (ANOVA/Tukey-Kramer) of relative amino acid contents in rosette leaves of soil-grown plants shown in Table 1 C.

Three comparisons: **Six comparisons:**

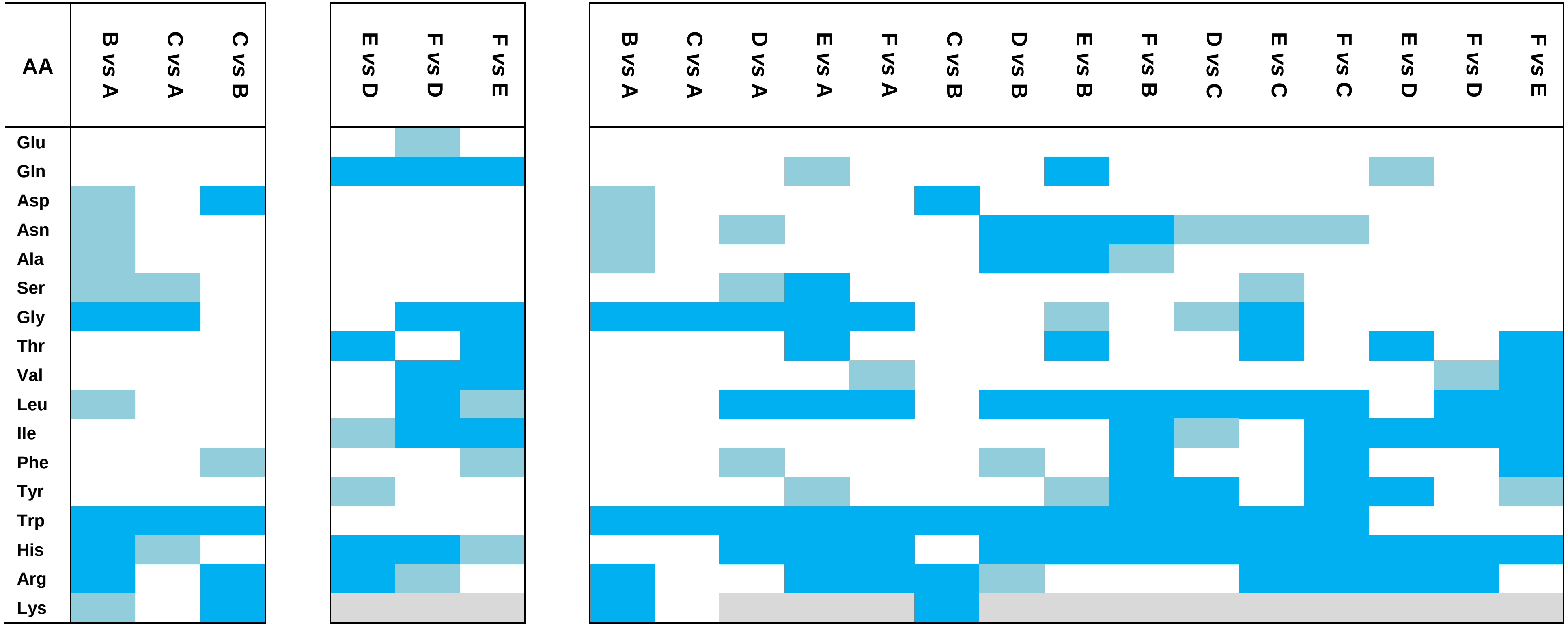

Supplemental Document 1, Table 1 E. Absolute Amino acid contents in rosette leaves of plants grown on ½ MS agar plates

**Amino acids**	**Col-0 (A)**	***cue1-6* (B)**	***lcd1-1* (C)**	**pOCA (D)**	***cue1-1* (E)**	***cue1-3* (F)**
				**($\mu mol \cdot g^{-1}$ fw)**		
**Glu**	0.836 ± 0.092	1.218 ± 0.151	1.217 ± 0.053	1.212 ± 0.117	2.597 ± 0.121	1.662 ± 0.031
**Gln**	3.216 ± 0.290	5.785 ± 0.442	4.934 ± 0.151	4.554 ± 0.207	9.780 ± 1.177	7.547 ± 0.221
**Asp**	0.818 ± 0.150	0.740 ± 0.099	0.963 ± 0.213	0.925 ± 0.190	0.835 ± 0.122	1.393 ± 0.353
**Asn**	1.221 ± 0.174	2.993 ± 0.338	1.795 ± 0.175	1.544 ± 0.076	4.383 ± 0.603	2.117 ± 0.065
**Ala**	0.516 ± 0.082	0.901 ± 0.097	0.547 ± 0.009	0.500 ± 0.032	1.264 ± 0.106	0.918 ± 0.029
**Ser**	2.403 ± 0.167	3.203 ± 0.268	2.874 ± 0.151	2.240 ± 0.009	4.073 ± 0.185	2.799 ± 0.095
**Gly**	4.617 ± 0.279	1.850 ± 0.286	3.605 ± 0.091	1.625 ± 0.188	2.340 ± 0.339	2.705 ± 0.287
**Thr**	0.396 ± 0.049	0.428 ± 0.030	0.435 ± 0.018	0.484 ± 0.032	0.560 ± 0.032	0.527 ± 0.016
**Val**	0.076 ± 0.011	0.091 ± 0.009	0.118 ± 0.006	0.126 ± 0.009	0.167 ± 0.005	0.130 ± 0.010
**Leu**	0.040 ± 0.014	0.023 ± 0.000	0.040 ± 0.008	0.038 ± 0.006	0.057 ± 0.008	0.039 ± 0.003
**Ile**	0.046 ± 0.011	0.032 ± 0.001	0.065 ± 0.005	0.076 ± 0.007	0.076 ± 0.012	0.066 ± 0.007
**Phe**	0.153 ± 0.019	0.031 ± 0.002	0.089 ± 0.003	0.079 ± 0.011	0.043 ± 0.006	0.079 ± 0.004
**Tyr**	0.013 ± 0.004	0.006 ± 0.000	0.026 ± 0.002	0.020 ± 0.004	0.028 ± 0.007	0.019 ± 0.003
**Trp**	0.013 ± 0.005	0.003 ± 0.000	0.017 ± 0.005	0.009 ± 0.003	0.013 ± 0.003	0.013 ± 0.004
**His**	0.058 ± 0.009	0.068 ± 0.008	0.113 ± 0.014	0.068 ± 0.005	0.135 ± 0.016	0.104 ± 0.014
**Arg**	1.156 ± 0.085	4.671 ± 0.302	3.326 ± 0.062	1.856 ± 0.064	8.661 ± 0.389	1.046 ± 0.122
**Lys**	0.263 ± 0.064	0.365 ± 0.008	0.164 ± 0.029	n.d	n.d.	n.d
**Total**	**15.8** ± **1.5**	**22.4** ± **2.0**	**20.3** ± **1.0**	**15.4** ± **1.0**	**35.0** ± **3.1**	**21.2** ± **1.3**

The data represent the mean ± SE of n = 5 replicates.

Supplemental Document 1, Table F. Statistical analysis (ANOVA/Tukey-Kramer) of absolute amino acid contents in rosette leaves of plants grown on ½ MS agar plates shown in Table 1 E.

Three comparisons:

Six comparisons:

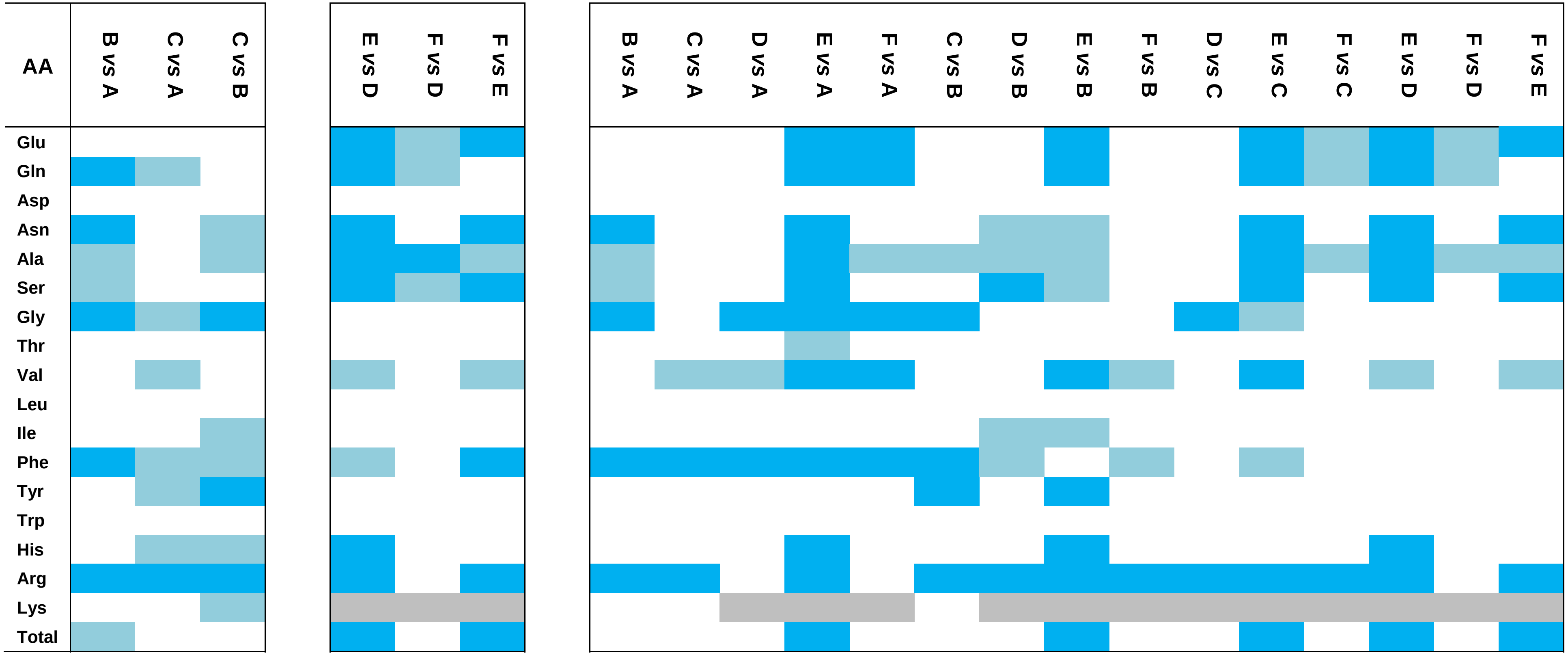

Supplemental Document 1, G. Relative Amino acid contents in rosette leaves of plants grown on ½ MS agar plates shown in Table 1 E.

Amino acids	Col-0 (A)	*cue1-6* (B)	*lcd1-1* (C)	pOCA (D)	*cue1-1* (E)	*cue1-3* (F)
				(%)		
**Glu**	5.28 ± 0.58	5.44 ± 0.68	5.99 ± 0.26	7.89 ± 0.76	7.42 ± 0.35	7.87 ± 0.15
**Gln**	20.30 ± 1.83	25.82 ± 1.97	24.27 ± 0.74	29.65 ± 1.35	27.93 ± 3.36	35.72 ± 1.05
**Asp**	5.16 ± 0.95	3.30 ± 0.44	4.74 ± 1.05	6.03 ± 1.24	2.38 ± 0.35	6.59 ± 1.67
**Asn**	7.71 ± 1.10	13.36 ± 1.51	8.83 ± 0.86	10.05 ± 0.49	12.52 ± 1.72	10.02 ± 0.31
**Ala**	3.26 ± 0.51	4.02 ± 0.43	2.69 ± 0.05	3.25 ± 0.21	3.61 ± 0.30	4.35 ± 0.14
**Ser**	**15.17 ± 1.05**	**14.30 ± 1.20**	**14.14 ± 0.74**	**14.59 ± 0.06**	**11.63 ± 0.53**	**13.25 ± 0.45**
**Gly**	29.14 ± 1.76	8.26 ± 1.28	17.74 ± 0.45	10.58 ± 1.22	6.68 ± 0.97	12.81 ± 1.36
**Thr**	2.50 ± 0.31	1.91 ± 0.14	2.14 ± 0.09	3.15 ± 0.21	1.60 ± 0.09	2.50 ± 0.07
**Val**	**0.48 ± 0.07**	**0.41 ± 0.04**	**0.58 ± 0.03**	**0.82 ± 0.06**	**0.48 ± 0.01**	**0.61 ± 0.05**
**Leu**	**0.25 ± 0.09**	**0.10 ± 0.00**	**0.20 ± 0.04**	**0.25 ± 0.04**	**0.16 ± 0.02**	**0.19 ± 0.01**
**Ile**	**0.29 ± 0.07**	**0.14 ± 0.01**	**0.32 ± 0.02**	**0.49 ± 0.05**	**0.22 ± 0.04**	**0.31 ± 0.03**
**Phe**	**0.96 ± 0.12**	**0.14 ± 0.01**	**0.44 ± 0.01**	**0.51 ± 0.07**	**0.12 ± 0.02**	**0.38 ± 0.02**
**Tyr**	**0.08 ± 0.03**	**0.03 ± 0.00**	**0.13 ± 0.01**	**0.13 ± 0.03**	**0.08 ± 0.02**	**0.09 ± 0.01**
**Trp**	**0.08 ± 0.03**	**0.01 ± 0.00**	**0.08 ± 0.02**	**0.06 ± 0.02**	**0.04 ± 0.01**	**0.06 ± 0.02**
**His**	0.37 ± 0.06	0.30 ± 0.04	0.55 ± 0.07	0.44 ± 0.03	0.39 ± 0.05	0.49 ± 0.07
**Arg**	**7.30 ± 0.54**	**20.84 ± 1.35**	**16.37 ± 0.30**	**12.09 ± 0.42**	**24.74 ± 1.11**	**4.95 ± 0.58**
**Lys**	1.66 ± 0.41	1.63 ± 0.04	0.81 ± 0.14	n.d.	n.d.	n.d.

The data represent the mean ± SE of n = 5 replicates.

Supplemental Document 1, Tabel 1 H. Statistical analysis (ANOVA/Tukey-Kramer) of relative amino acid contents in rosette leaves of plants grown on ½ MS agar shown in Table 1 G.

Three comparisons: **Six comparisons:**

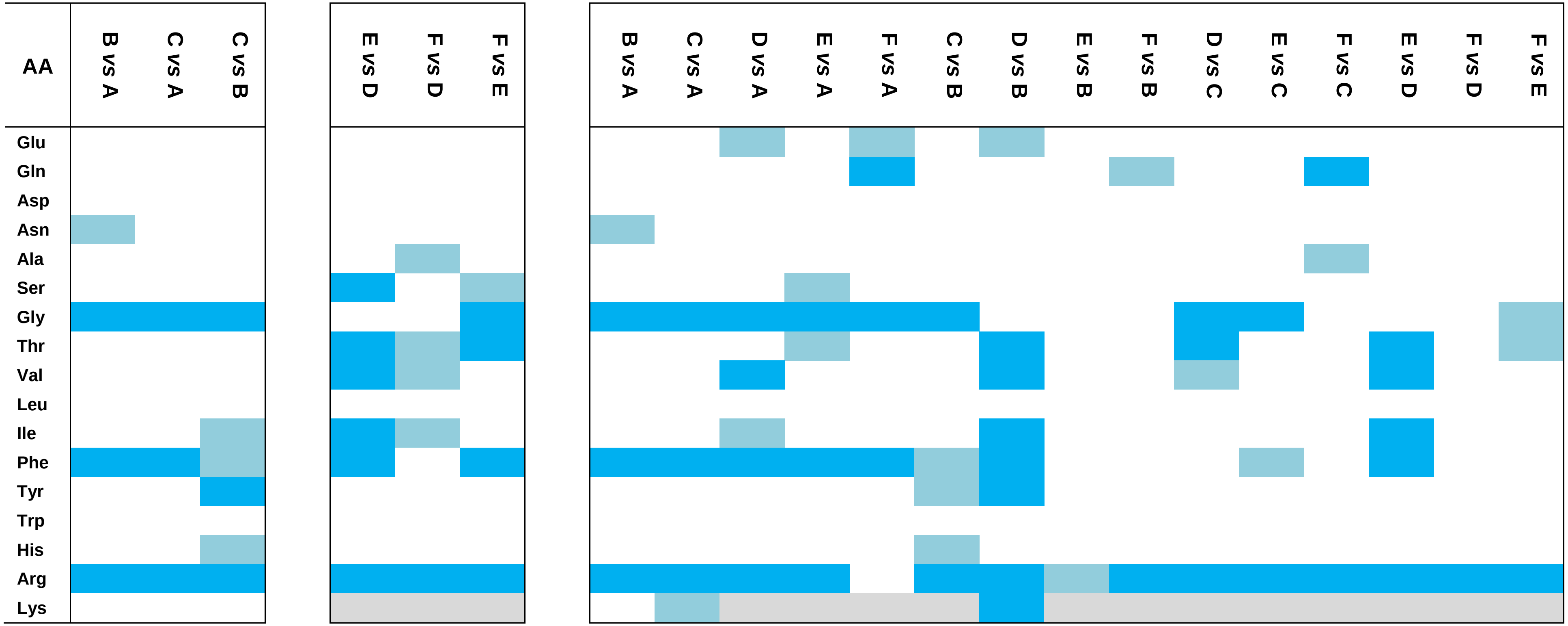

Supplemental Document 1, Table 1 I. Absolute Amino acid contents in roots of plants grown on ½ MS agar plates

**Amino acids**	**Col-0 (A)**	***cue1-6* (B)**	***lcd1-1* (C)**	**pOCA (D)**	***cue1-1* (E)**	***cue1-3* (F)**
			**(µmol·g^{-1} fw)**			
**Glu**	0.450 ± 0.032	0.545 ± 0.012	1.142 ± 0.050	0.289 ± 0.014	0.461 ± 0.056	0.321 ± 0.058
**Gln**	3.166 ± 0.179	5.660 ± 0.345	4.978 ± 0.267	2.312 ± 0.095	5.259 ± 0.476	2.841 ± 0.248
**Asp**	0.529 ± 0.035	0.450 ± 0.081	0.846 ± 0.091	0.413 ± 0.029	0.590 ± 0.048	0.581 ± 0.057
**Asn**	1.174 ± 0.113	2.610 ± 0.134	1.590 ± 0.158	0.562 ± 0.060	1.776 ± 0.035	0.703 ± 0.023
**Ala**	0.642 ± 0.051	0.904 ± 0.060	0.518 ± 0.056	0.357 ± 0.022	0.844 ± 0.076	0.425 ± 0.046
**Ser**	**1.017 ± 0.082**	**3.893 ± 0.208**	**1.459 ± 0.063**	**0.872 ± 0.120**	**2.114 ± 0.066**	**1.019 ± 0.040**
**Gly**	0.292 ± 0.034	0.550 ± 0.010	0.371 ± 0.022	0.238 ± 0.028	0.570 ± 0.049	0.383 ± 0.038
**Thr**	0.593 ± 0.030	0.942 ± 0.096	0.758 ± 0.049	0.421 ± 0.026	0.824 ± 0.069	0.704 ± 0.045
**Val**	**0.163 ± 0.009**	**0.239 ± 0.026**	**0.200 ± 0.015**	**0.133 ± 0.012**	**0.216 ± 0.016**	**0.256 ± 0.020**
**Leu**	**0.080 ± 0.017**	**0.159 ± 0.046**	**0.140 ± 0.019**	**0.063 ± 0.010**	**0.094 ± 0.013**	**0.173 ± 0.019**
**Ile**	**0.084 ± 0.006**	**0.178 ± 0.027**	**0.105 ± 0.016**	**0.058 ± 0.007**	**0.109 ± 0.017**	**0.151 ± 0.014**
**Phe**	**0.039 ± 0.002**	**0.061 ± 0.005**	**0.053 ± 0.004**	**0.047 ± 0.002**	**0.057 ± 0.006**	**0.099 ± 0.010**
**Tyr**	**0.021 ± 0.002**	**0.031 ± 0.006**	**0.025 ± 0.004**	**0.009 ± 0.002**	**0.020 ± 0.002**	**0.035 ± 0.002**
**Trp**	**0.183 ± 0.036**	**0.303 ± 0.053**	**0.077 ± 0.023**	**0.056 ± 0.001**	**0.190 ± 0.036**	**0.089 ± 0.016**
**His**	0.134 ± 0.012	0.257 ± 0.014	0.180 ± 0.013	0.090 ± 0.011	0.167 ± 0.015	0.138 ± 0.010
**Arg**	**1.235 ± 0.116**	**1.332 ± 0.075**	**0.410 ± 0.121**	**0.374 ± 0.079**	**0.609 ± 0.225**	**0.440 ± 0.116**
**Lys**	0.394 ± 0.021	0.554 ± 0.013	0.448 ± 0.097	n.d.	n.d.	n.d.
**Total**	**10.2** ± **0.8**	**18.7** ± **1.2**	**13.3** ± **1.1**	**6.3** ± **0.5**	**13.9** ± **1.2**	**8.4** ± **0.8**

The data represent the mean ± SE of n = 3-5 replicates.

Supplemental Document 1, Table 1 J. Statistical analysis (ANOVA/Tukey-Kramer) of absolute amino acid contents in roots of plants grown on ½ MS agar plates shown in Table 1 I.

Three comparisons:

AA	B vs A	C vs A	C vs B
Glu			
Gln			
Asp			
Asn			
Ala			
Ser			
Gly			
Thr			
Val			
Leu			
Ile			
Phe			
Tyr			
Trp			
His			
Arg			
Lys			
Total			

E vs D	F vs D	F vs E

Six comparisons:

B vs A	C vs A	D vs A	E vs A	F vs A	C vs B	D vs B	E vs B	F vs B	D vs C	E vs C	F vs C	E vs D	F vs D	F vs E

Supplemental Document 1, Table 1 K. Relative amino acid contents in roots of plants grown on ½ MS agar calculated from Table 1 I.

Amino acids	Col-0 (A)	*cue1-6* (B)	*lcd1-1* (C)	pOCA (D)	*cue1-1* (E)	*cue1-3* (F)
				(%)		
**Glu**	4.41 ± 0.31	2.92 ± 0.06	8.59 ± 0.37	4.60 ± 0.22	3.32 ± 0.41	3.84 ± 0.69
**Gln**	31.05 ± 1.75	30.32 ± 1.85	37.43 ± 2.01	36.73 ± 1.51	37.83 ± 3.42	33.99 ± 2.96
**Asp**	5.19 ± 0.34	2.41 ± 0.44	6.36 ± 0.68	6.57 ± 0.46	4.24 ± 0.34	6.95 ± 0.69
**Asn**	11.52 ± 1.11	13.98 ± 0.72	11.95 ± 1.19	8.93 ± 0.95	12.78 ± 0.25	8.42 ± 0.28
**Ala**	6.30 ± 0.50	4.84 ± 0.32	3.89 ± 0.42	5.68 ± 0.34	6.07 ± 0.55	5.08 ± 0.55
**Ser**	9.97 ± 0.80	20.85 ± 1.11	10.97 ± 0.48	13.86 ± 1.91	15.21 ± 0.47	12.19 ± 0.48
**Gly**	2.86 ± 0.33	2.95 ± 0.05	2.79 ± 0.17	3.77 ± 0.45	4.10 ± 0.35	4.58 ± 0.46
**Thr**	5.82 ± 0.30	5.05 ± 0.51	5.70 ± 0.37	6.69 ± 0.41	5.93 ± 0.50	8.42 ± 0.54
**Val**	**1.60 ± 0.09**	**1.28 ± 0.14**	**1.50 ± 0.11**	**2.11 ± 0.19**	**1.55 ± 0.12**	**3.07 ± 0.24**
**Leu**	**0.78 ± 0.17**	**0.85 ± 0.25**	**1.05 ± 0.15**	**0.99 ± 0.16**	**0.67 ± 0.09**	**2.06 ± 0.22**
**Ile**	**0.83 ± 0.06**	**0.95 ± 0.15**	**0.79 ± 0.12**	**0.91 ± 0.12**	**0.79 ± 0.12**	**1.81 ± 0.17**
**Phe**	**0.38 ± 0.02**	**0.33 ± 0.03**	**0.39 ± 0.03**	**0.74 ± 0.03**	**0.41 ± 0.05**	**1.19 ± 0.13**
**Tyr**	**0.21 ± 0.02**	**0.17 ± 0.03**	**0.19 ± 0.03**	**0.14 ± 0.04**	**0.15 ± 0.01**	**0.42 ± 0.02**
**Trp**	**1.80 ± 0.35**	**1.62 ± 0.28**	**0.58 ± 0.17**	**0.90 ± 0.01**	**1.36 ± 0.26**	**1.07 ± 0.19**
**His**	1.31 ± 0.12	1.38 ± 0.07	1.36 ± 0.10	1.43 ± 0.17	1.20 ± 0.11	1.65 ± 0.12
**Arg**	**12.11 ± 1.13**	**7.14 ± 0.40**	**3.08 ± 0.91**	**5.95 ± 1.26**	**4.38 ± 1.62**	**5.26 ± 1.39**
**Lys**	3.86 ± 0.20	2.97 ± 0.07	3.37 ± 0.73	n.d.	n.d.	n.d.

The data represent the mean ± SE of n = 3-5 replicates.

Supplemental Document 1, Table 1 L. Statistical analysis (ANOVA/Tukey-Kramer) of relative amino acid contents in roots of plants grown on ½ MS agar shown in Table 1 K.

Three comparisons: **Six comparisons:**

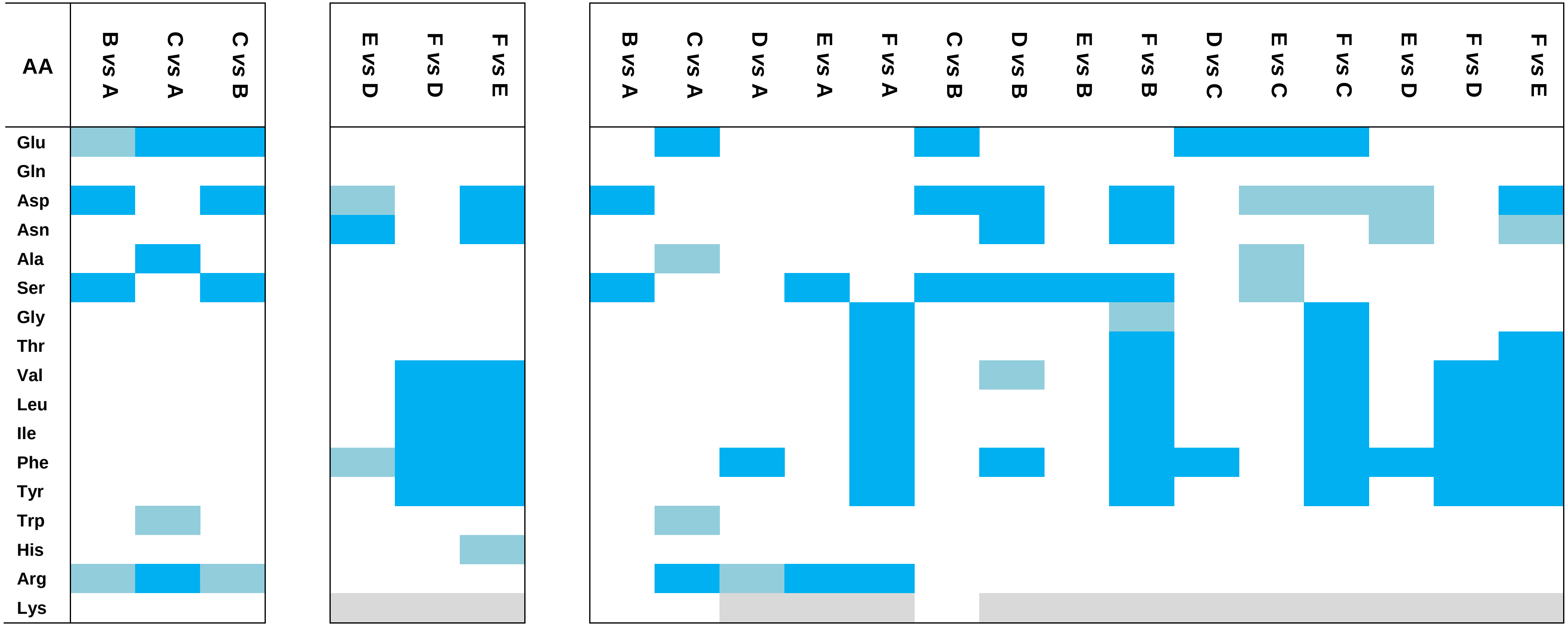

Supplemental Document 1, Table 2 A. Amino acid contents in rosette leaves of plants grown on ½MS aga in the absence (A-C) or presence of AAA (D-F) or tZ (G-I).

**Amino acids**	**Col-0 control (A)**	***cue1-6 control* (B)**	***lcd1-1 control* (C)**	**Col-0 AAA (D)**	***cue1-6 AAA* (E) (μmol·g^{-1} fw)**	***lcd1 AAA* (F)**	**Col-0 tZ (G)**	***cue1-6 tZ* (H)**	***lcd1 tZ* (I)**
**Glu**	1.31 ± 0.01	1.94 ± 0.03	1.42 ± 0.02	2.01 ± 0.05	3.13 ± 0.05	1.63 ± 0.01	2.12 ± 0.06	1.98 ± 0.03	1.60 ± 0.10
**Gln**	10.55 ± 0.38	18.32 ± 0.56	13.31 ± 0.16	12.93 ± 0.19	21.88 ± 0.52	11.46 ± 0.13	20.32 ± 0.26	23.28 ± 0.25	20.25 ± 0.17
**Asp**	0.77 ± 0.02	0.60 ± 0.03	1.08 ± 0.02	1.85 ± 0.03	1.37 ± 0.04	1.41 ± 0.01	1.30 ± 0.07	0.73 ± 0.02	1.06 ± 0.10
**Asn**	5.06 ± 0.09	9.09 ± 0.24	5.59 ± 0.07	3.07 ± 0.04	5.70 ± 0.15	2.53 ± 0.03	5.02 ± 0.02	5.35 ± 0.07	7.88 ± 0.08
**Ala**	0.70 ± 0.01	1.48 ± 0.03	0.86 ± 0.02	0.89 ± 0.02	2.04 ± 0.06	0.82 ± 0.01	1.21 ± 0.04	1.16 ± 0.03	0.82 ± 0.03
**Ser**	1.14 ± 0.02	1.71 ± 0.04	1.08 ± 0.06	1.92 ± 0.04	4.26 ± 0.12	1.70 ± 0.05	2.48 ± 0.09	3.07 ± 0.06	1.86 ± 0.05
**Gly**	0.65 ± 0.05	0.27 ± 0.04	0.30 ± 0.04	0.46 ± 0.01	1.39 ± 0.04	0.42 ± 0.04	0.66 ± 0.08	0.65 ± 0.00	0.56 ± 0.03
**Thr**	0.44 ± 0.01	0.47 ± 0.02	0.46 ± 0.01	0.58 ± 0.01	0.68 ± 0.04	0.49 ± 0.01	0.73 ± 0.03	0.50 ± 0.02	0.47 ± 0.02
**Val**	0.24 ± 0.00	0.29 ± 0.01	0.25 ± 0.01	0.28 ± 0.01	0.37 ± 0.01	0.26 ± 0.01	0.27 ± 0.01	0.21 ± 0.01	0.21 ± 0.01
**Leu**	0.16 ± 0.01	0.17 ± 0.02	0.15 ± 0.00	0.08 ± 0.00	0.13 ± 0.01	0.07 ± 0.00	0.19 ± 0.01	0.13 ± 0.00	0.12 ± 0.00
**Ile**	0.18 ± 0.00	0.22 ± 0.00	0.19 ± 0.01	0.09 ± 0.00	0.12 ± 0.01	0.08 ± 0.00	0.17 ± 0.01	0.13 ± 0.00	0.15 ± 0.01
**Phe**	0.09 ± 0.00	0.11 ± 0.00	0.10 ± 0.00	7.36 ± 0.10	7.92 ± 0.19	6.40 ± 0.07	0.13 ± 0.01	0.08 ± 0.00	0.09 ± 0.00
**Tyr**	0.06 ± 0.00	0.08 ± 0.00	0.06 ± 0.01	7.58 ± 0.11	7.68 ± 0.18	6.62 ± 0.06	0.07 ± 0.00	0.04 ± 0.00	0.03 ± 0.00
**Trp**	0.03 ± 0.00	0.05 ± 0.01	0.03 ± 0.00	5.58 ± 0.08	7.54 ± 0.18	4.86 ± 0.05	0.09 ± 0.01	0.08 ± 0.01	0.06 ± 0.00
**His**	0.07 ± 0.00	0.11 ± 0.01	0.10 ± 0.01	0.10 ± 0.00	0.16 ± 0.01	0.11 ± 0.01	0.12 ± 0.00	0.08 ± 0.00	0.09 ± 0.00
**Arg**	2.07 ± 0.08	7.78 ± 0.20	1.31 ± 0.02	2.20 ± 0.03	3.99 ± 0.12	0.95 ± 0.00	2.21 ± 0.05	2.99 ± 0.05	0.71 ± 0.01
**Lys**									
**Total**	**23.52 ± 0.69**	**42.68 ± 1.24**	**26.29 ± 0.48**	**46.97 ± 0.73**	**68.35 ± 1.73**	**39.82 0.53**	**37.08 ± 0.74**	**40.45 ± 0.55**	**35.95 ± 0.60**

The data represent the mean ± SE of n = 5 replicates.

Supplemental Document 1, Table 2 B. Statistical analysis (ANOVA/Tukey-Kramer) of absolute amino acid contents in leaves grown on ½ MS agar or ½ MS agar supplemented with a cocktail of AAA as shown in Table 2 A.

AA	B vs A	C vs A	D vs A	E vs A	F vs A	C vs B	D vs B	E vs B	F vs B	D vs C	E vs C	F vs C	E vs D	F vs D	F vs E
Glu															
Gln															
Asp															
Asn															
Ala															
Ser															
Gly															
Thr															
Val															
Leu															
Ile															
Phe															
Tyr															
Trp															
His															
Arg															
Total															

Supplemental Document 1, Table 2 C. Statistical analysis (ANOVA/Tukey-Kramer) of absolute amino acid contents in leaves grown on ½ MS agar or ½ MS agar supplemented with tZ as shown in Table 2 A.

AA	B vs A	C vs A	G vs A	H vs A	I vs A	C vs B	G vs B	H vs B	I vs B	G vs C	H vs C	I vs C	H vs G	I vs G	I vs H
Glu															
Gln															
Asp															
Asn															
Ala															
Ser															
Gly															
Thr															
Val															
Leu															
Ile															
Phe															
Tyr															
Trp															
His															
Arg															
Total															

Supplemental Document 1, Table 2 D. Statistical analysis (ANOVA/Tukey-Kramer) of absolute amino acid contents in leaves grown on ½ MS agar supplemented with a cocktail of AAA or ½ MS agar supplemented with tZ as shown in Table 2 A.

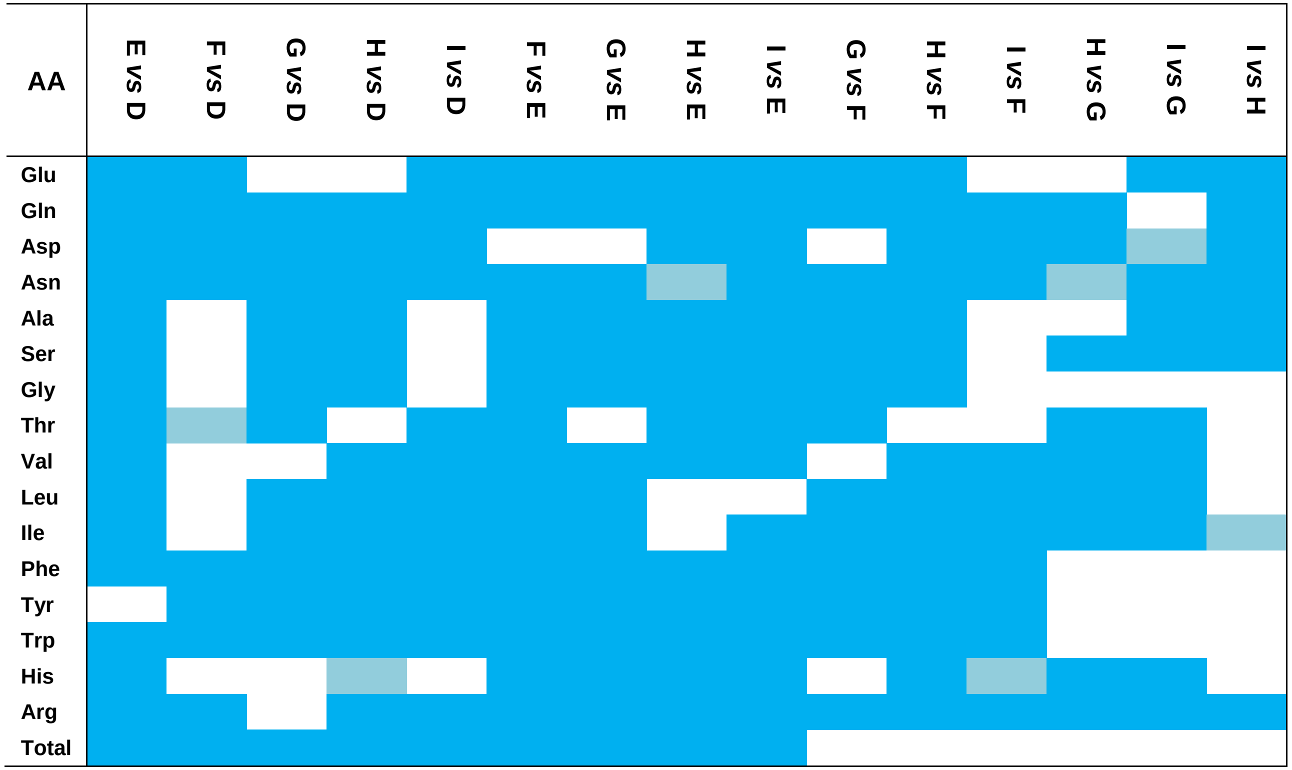

Supplemental Figures

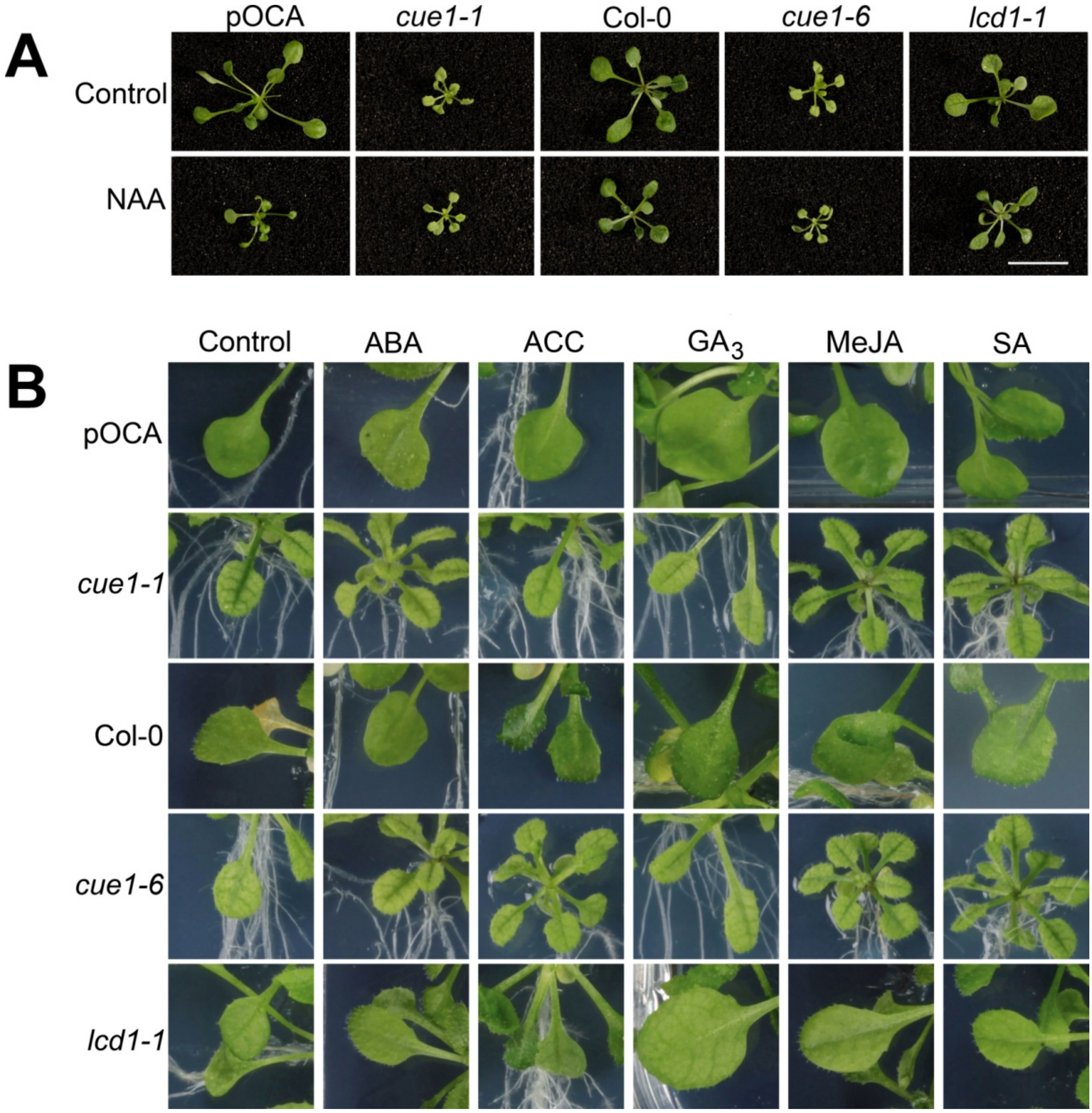

Supplemental Figure 1. Effect of phytohormone treatment on the leaf phenotypes of *cue1* and *lcd1-1* compared to wild-type or control plants.

Phytohormones were applied at final concentrations of 10 µM. The plants were grown for three weeks on ½MS agar and were then transferred for seven days to ½MS agar supplemented with the effectors. In a first set of experiments, the impact of NAA feeding on the phenotype of *cue1* and *lcd1* was investigated (**A**). In a second set of experiments, various phytohormones were applied and their effect on the reticulate leaf phenotype of *cue1* and *lcd1* was analysed in comparison to wild-type or control plants (**B**). The bar in (**A**) represents a length of 1 cm.

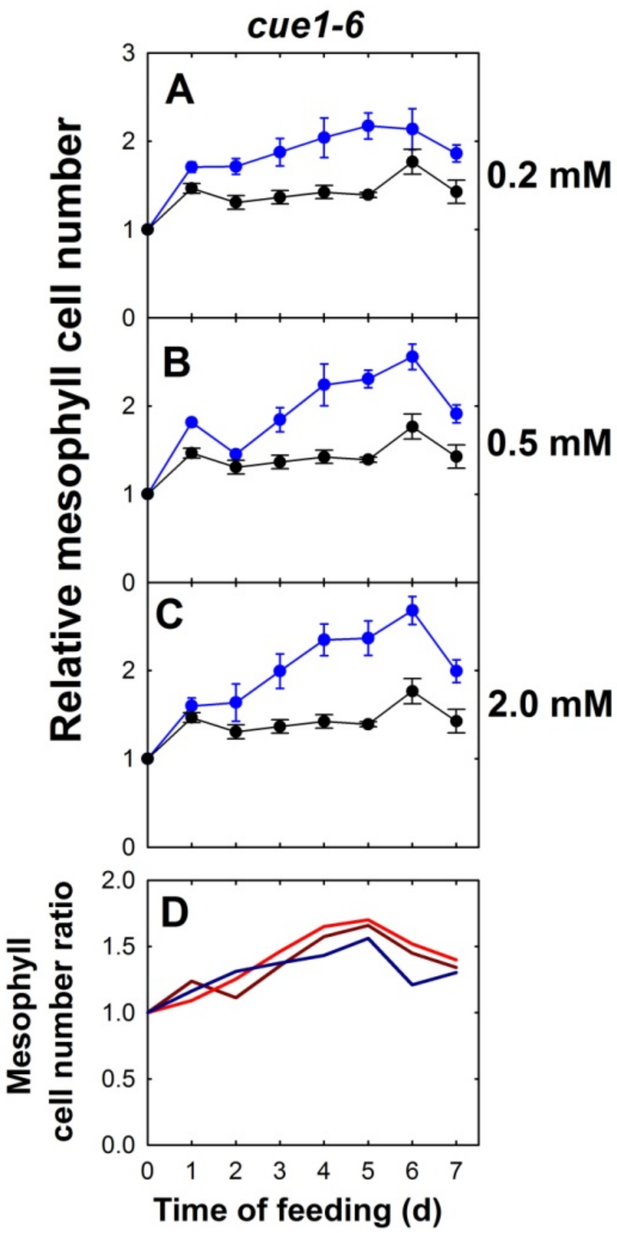

Supplemental Figure 2. Effects of increasing AAA concentrations on relative mesophyll cell numbers.

In **A-C** the time-dependent development of relative mesophyll cell densities in *cue1* (blue lines and symbols) is shown compared to the wild type (black lines and symbols). In **D** the cell number ratios (treated/untreated) is shown for 0.2 mM (blue line), 0.5 mM (dark red line), and 2 mM (light red line) of each AAA in the cocktail. The initial cell density was about 100, 60, or 86 cells per 0.05 mm^2 in leaves of Col-0, *cue1-6*, or *lcd1-1*, respectively. The data represent the mean ± SE of n = 10 measurements per time point and condition.

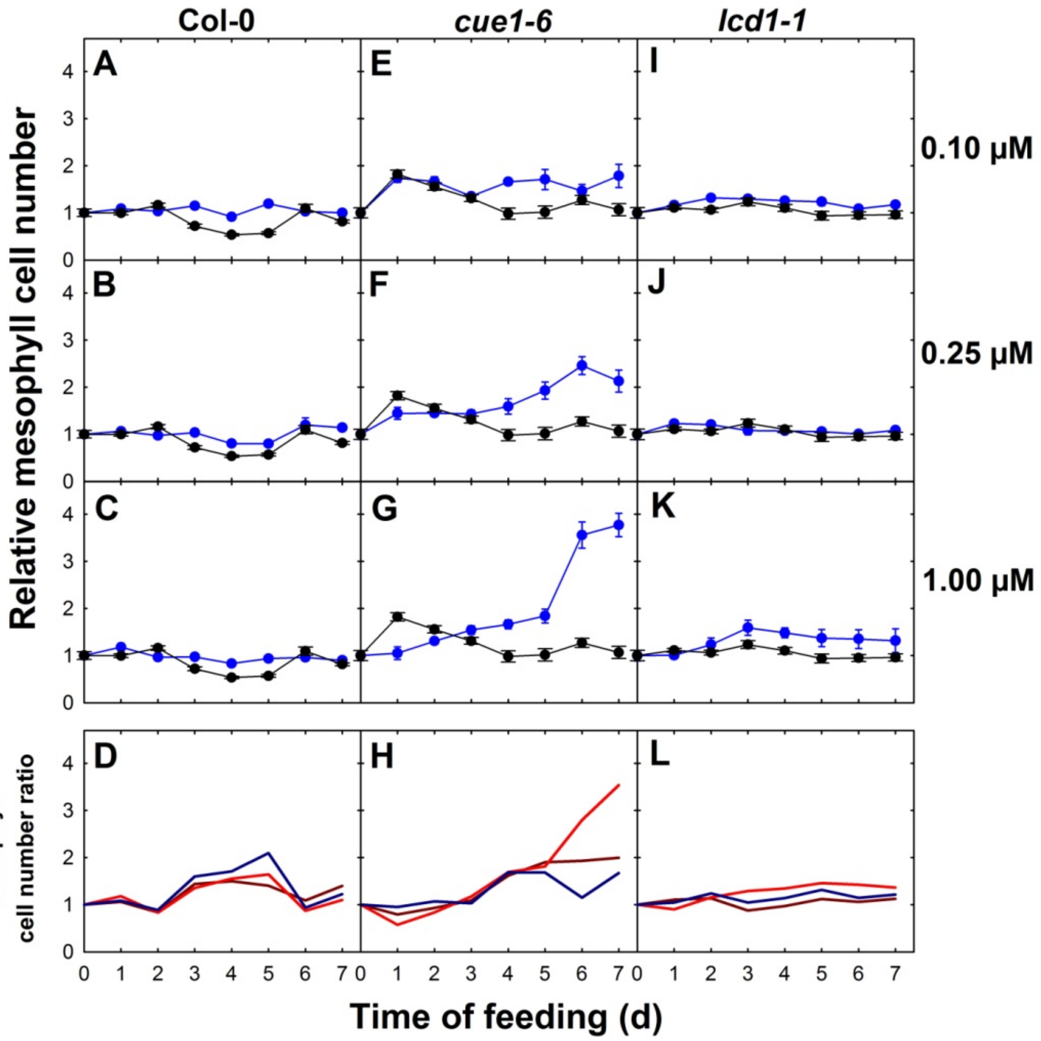

Supplemental Figure 3. Effects of increasing *tZ* concentrations on relative mesophyll cell numbers.

The time-dependent development of relative mesophyll cell densities are shown for Col-0 (**A-C**), *cue1-6* (**E-G**), and *lcd1-1* (**I-K**). The blue and black lines and symbols represent treated and untreated control plants, respectively. In **D-H** the cell number ratios (treated/untreated) is shown for 0.1 µM (blue line), 0.25 µM (dark red line), and 1.0 µM (light red line) of *tZ*. The initial cell density was about 100, 60, or 86 cells per 0.05 mm^2 in leaves of Col-0, *cue1-6*, or *lcd1-1*, respectively. The data represent the mean ± SE of n = 10 measurements per time point and condition.

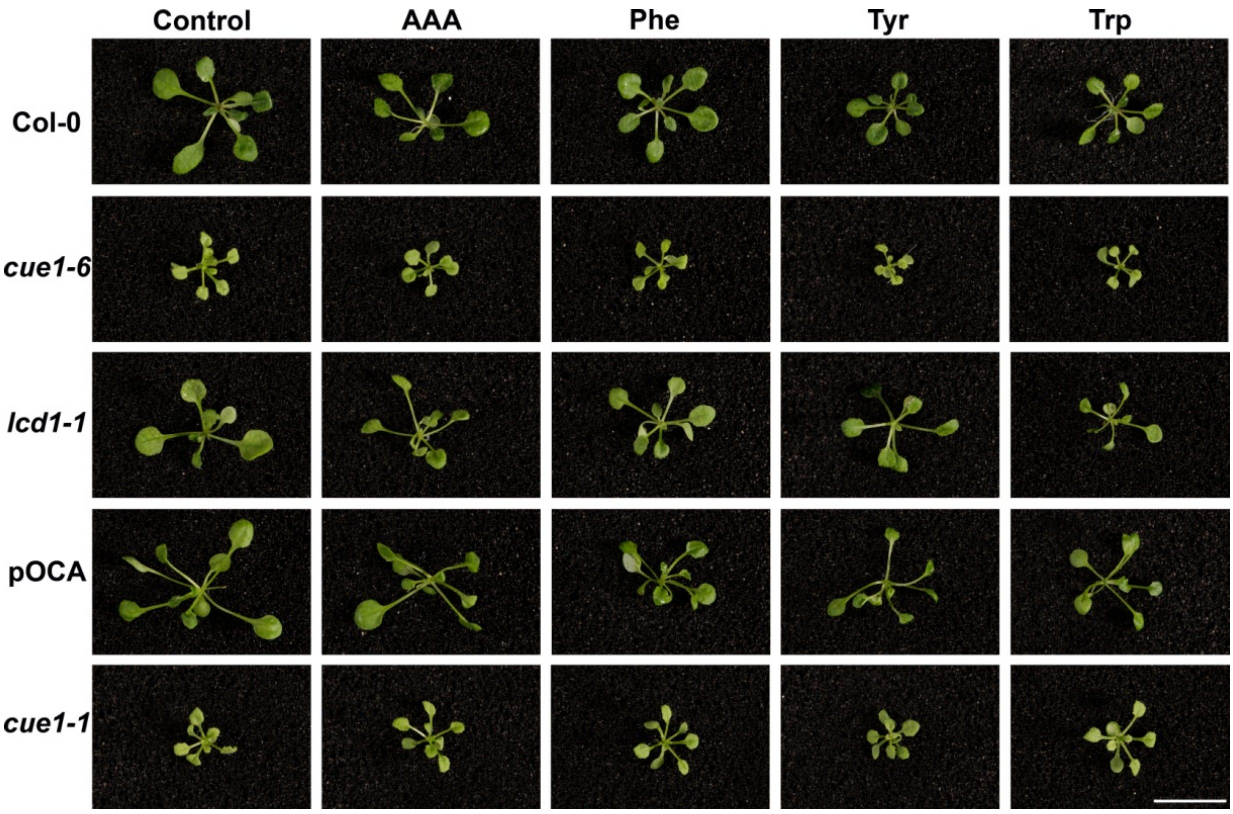

Supplemental Figure 4. Rosette phenotypes after seven days of feeding with individual AAA or a cocktail of AAA.

Col-0, pOCA, *cue1-1*, *cue1-6,* and *lcd1-1* were fed with individual AAA (2 mM each) or a cocktail of AAA and compared to unfed control plants. The plants were grown for three weeks on ½MS agar and were then transferred to ½MS agar supplemented with the effectors. The bar represents a length of 1 cm.

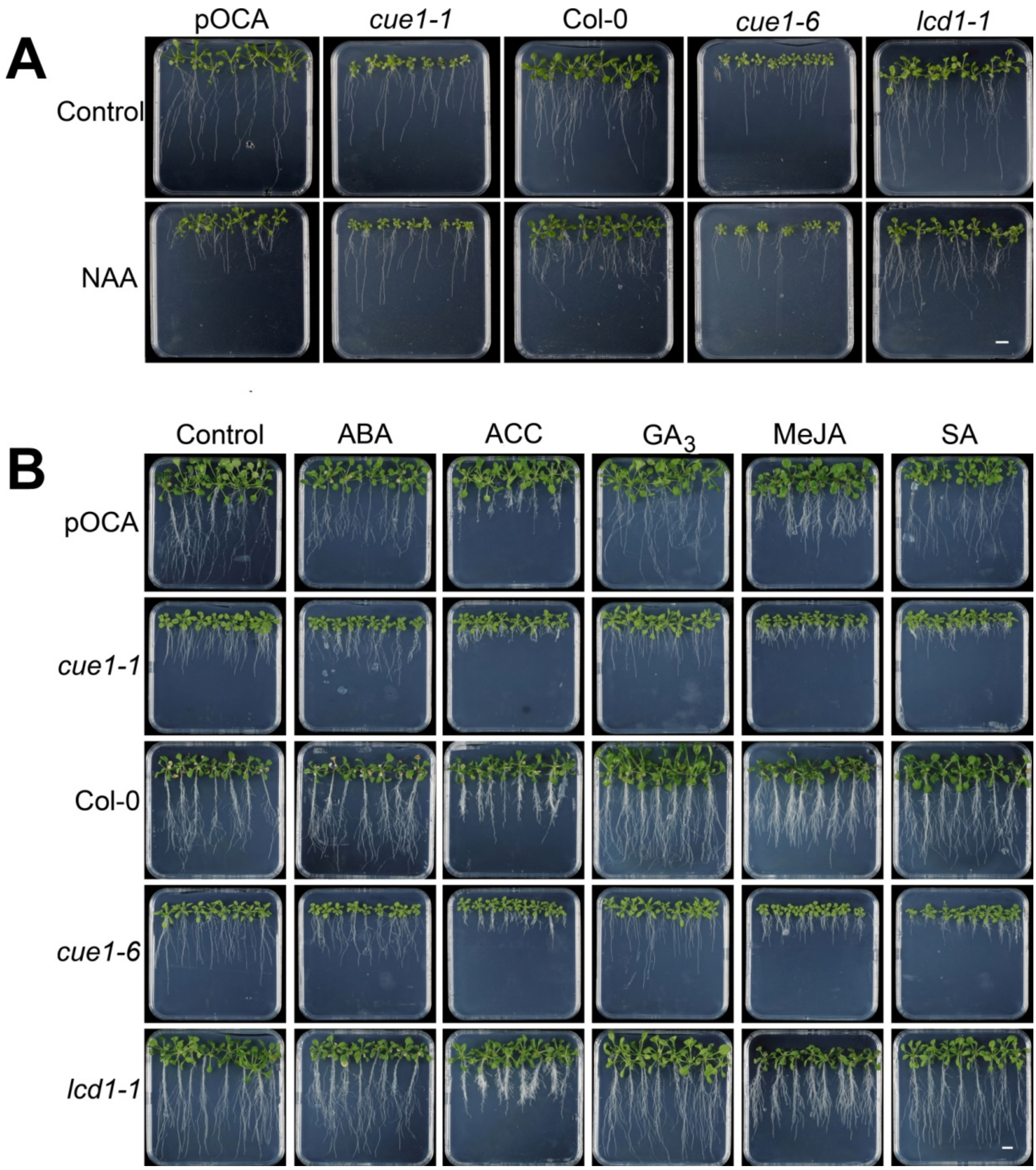

Supplemental Figure 5. Root phenotypes after seven days of feeding with individual phytohormones.

Col-0. pOCA, *cue1-1*, *cue1-6* and *lcd1-1* were fed with individual phytohormones (10 µM each) and compared to unfed control plants. The plants were grown for three weeks on ½MS agar and were then transferred to ½MS agar supplemented with the effectors. In a first set of experiments, the impact of NAA feeding on the phenotype of *cue1* and *lcd1* was investigated (**A**). In a second set of experiments, various phytohormones were applied and their effect on the reticulate leaf phenotype of *cue1* and *lcd1* was analysed in comparison to wild-type or control plants (**B**). The bars in (**A**) and (**B**) represent the length of 1 cm.
